# Schottky-driven interfacial design of Bi_2_MoO_6_/Ti_3_C_2_T_x_ heterostructure for boosted piezocatalytic hydrogen evolution

**DOI:** 10.1038/s43246-026-01168-z

**Published:** 2026-05-06

**Authors:** Rahil Changotra, Jie Yang, Mita Dasog, Quan Sophia He

**Affiliations:** 1 Department of Engineering, Faculty of Agriculture, Dalhousie University, Truro, NS Canada; 2 Institute of Oceanography, College of Geography and Oceanography, Minjiang University, Fuzhou, China; 3 Department of Chemistry, Dalhousie University, Halifax, NS Canada; 4 Department of Civil and Resource Engineering, Dalhousie University, Halifax, NS Canada

**Keywords:** Heterogeneous catalysis, Materials for energy and catalysis

## Abstract

Piezoelectric semiconductor catalysis is gaining attention as a strategy to convert mechanical energy into chemical energy for sustainable hydrogen production. Similar to photocatalysis, piezocatalysis involves the generation, separation, migration, and surface reaction of piezo-induced charge carriers. Here, we report the rational design of Bi_2_MoO_6_/Ti_3_C_2_T_x_ piezocatalysts synthesized via electrostatic self-assembly and evaluate their hydrogen evolution performance. The optimized heterostructure achieves a hydrogen evolution rate of 1.99 ${mmol}{g}^{-1}{h}^{-1}$, which is 2.75 and 5.78 times higher than pristine Bi_2_MoO_6_ and nanolayered Ti_3_C_2_T_x_, respectively. Experimental characterization combined with density functional theory calculations demonstrates that the heterointerface facilitates rapid electron transfer and enhances the intrinsic piezoelectric response. Furthermore, the interface reduces the hydrogen adsorption energy barrier and improves Gibbs free energy for water splitting, leading to enhanced charge separation and suppressed carrier recombination. A Schottky junction-based mechanism is proposed to explain directional charge transport and surface redox reactions under mechanical stimulation, providing new design insights for high-efficiency piezocatalysts driven by low-intensity mechanical energy.

## Abstract (web summary)

Piezoelectric semiconductor catalysts have been shown to be capable of converting mechanical energy into chemical energy for sustainable hydrogen production. Here, Bi₂MoO₆/Ti₃C₂Tx piezocatalysts are synthesized via electrostatic self-assembly, and their hydrogen evolution performance is evaluated.

## Introduction

The escalating global demand for energy, coupled with the environmental consequences of fossil fuel combustion, has intensified the need for clean and sustainable energy alternatives. Among various chemical energy carriers, hydrogen (H_2_) stands out due to its high gravimetric energy density and zero-carbon emission upon utilization, making it a key player in the future energy economy.^1,2^ Solar-driven photocatalytic water splitting offers a compelling route for green hydrogen production, leveraging abundant solar energy to generate chemical fuels.^3,4^ However, the efficiency of this process critically depends on the properties of the semiconductor photocatalyst, including its light-harvesting capability, charge carrier mobility and separation, and surface reaction kinetics.^5,6^ Despite decades of progress, many photocatalysts suffer from intrinsic limitations such as narrow spectral absorption, rapid electron–hole recombination, and sluggish surface redox reactions. Beyond solar energy, mechanical energy can be harnessed for catalysis via piezoelectric semiconductor materials, a process termed “piezocatalysis”.^7,8^ Unlike photocatalysis, piezocatalysis involves mechanical vibrations to induce piezo-induced carriers (electrons and holes) in piezoelectric materials, thereby initiating redox reactions. This energy conversion relies on the synergistic interaction between piezoelectric polarization and interfacial charge transfer, where the internal electric field generated under mechanical stress acts as the driving force for carrier separation and migration.^8,9^ Thus, advancing high-performance piezocatalysts and developing strategies to enhance their catalytic efficiency are crucial for unlocking the full potential of this emerging technology.

Recent research has seen a surge in the development of piezocatalytic materials, attributed to their large surface area, high carrier mobility, excellent strain responsiveness, and intrinsic piezoelectric properties. In the past, piezocatalytic materials such as g-C_3_N_4_,^10^ MoS_2_,^11^ ZnO,^12^ SnSe,^13^ Bi_2_WO_6_,^14^ and BiFeO_3_^5^ have emerged as promising candidates for piezocatalytic applications. In particular, bismuth molybdate (Bi_2_MoO_6_) with a layered structure has attracted attention due to its tunable electronic configuration, high stability, and capacity for strain relaxation during catalysis.^15,16^ Recent work by Zhang et al.^17^ demonstrated that pristine Bi_2_MoO_6_ could facilitate hydrogen evolution under ultrasonic-induced vibrational energy at a rate of 16.36 $\mu {mol}{g}^{-1}{h}^{-1}$, confirming its piezocatalytic potential. Nevertheless, its low piezocatalytic efficiency for H_2_ evolution is hindered by intrinsic drawbacks, including rapid recombination of charge carriers, sluggish surface kinetics, and a limited number of active sites. Addressing these challenges is essential for the rational design of more efficient piezocatalytic systems for H_2_ evolution. Beyond Bi_2_MoO_6_, a broader class of polar layered bismuth-containing compounds, including BiOX (X = Cl, Br, I) and Aurivillius-type bismuth oxides, has recently emerged as promising piezocatalysts for mechanically driven hydrogen evolution. These materials typically feature alternating charged layers and stereochemically active Bi^3+^ 6s^2^ lone pairs, which induce intrinsic polarization and enable effective strain-responsive charge separation under vibrational excitation.^18–20^ Such structural characteristics provide a favorable platform for piezocatalysis and suggest that performance limitations in pristine layered bismuth oxides can be overcome through rational structural and interfacial engineering.

In parallel, 2D transition metal carbides and nitrides, known as MXenes, have gained tremendous attention for their outstanding electrical conductivity, high specific surface area, and mechanical robustness. Among them, Ti_3_C_2_, a titanium-based MXene, is particularly attractive due to its metallic conductivity and ability to facilitate rapid electron transport. MXenes have demonstrated their efficacy in diverse energy-related applications, including photocatalysis, electrochemical energy storage, sensing, and photoelectrochemical systems.^21–23^ Their function as cocatalysts is especially promising, as they can effectively promote interfacial charge separation when integrated with conventional semiconductors such as TiO_2_,^24^ ZnO,^25^ La_2_Ti_2_O_7_,^26^ and MoS_2_.^27^ However, the application of Ti_3_C_2_ MXene in piezocatalysis remains largely unexplored, opening new opportunities for its integration into mechanical-to-chemical energy conversion platforms.

The integration of layered Ti_3_C_2_T_X_ to tailor the structural and catalytic properties of Bi_2_MoO_6_ offers significant potential, yet its application in piezocatalytic hydrogen production remains largely underexplored, thus emphasizing the importance of further in-depth investigation of the heterostructure in this emerging field. The layered Ti_3_C_2_T_x_ structure can theoretically act as a robust support for the nanosheet architecture of Bi_2_MoO_6_. By tailoring its surface terminal groups, the work function and piezoelectric properties of Ti_3_C_2_T_x_ can be modulated, facilitating the formation of a Schottky junction with Bi_2_MoO_6_ and thereby enhancing its catalytic performance.^28,29^ Based on this concept, we report on the rational design and fabrication of a series of Bi_2_MoO_6_/Ti_3_C_2_T_X_ heterostructure piezocatalysts to drive H_2_ evolution via ultrasonic mechanical vibrations. We synthesized three-dimensional (3D) hierarchical porous Bi_2_MoO_6_ microspheres, which were fabricated onto nanolayered Ti_3_C_2_T_x_ through an electrostatic self-assembly approach. Comprehensive characterization, including piezoresponse force microscopy (PFM) and ultraviolet photoelectron spectroscopy (UPS), confirms the formation of Schottky junctions at the Bi_2_MoO_6_/Ti_3_C_2_T_X_ interface, facilitating the development of an internal electric field under ultrasonic vibration that promotes the directional migration of piezo-induced carriers. By leveraging a cocatalyst engineering approach and mechanical vibrational energy, we aim to elucidate the underlying piezocatalytic mechanism through experimental and theoretical calculation methods. Our experimental results reveal that the Bi_2_MoO_6_/Ti_3_C_2_T_X_ composite exhibits a substantially enhanced H_2_ evolution rate compared to pristine Bi_2_MoO_6_ and several reported piezocatalysts reported in the literature. This work not only introduces a novel application of MXene materials in the piezocatalytic domain but also provides new insights into the interfacial mechanisms supporting mechanical-to-chemical energy conversion. Our findings provide a comprehensive approach for designing high-efficiency piezocatalytic systems and contribute to the broader pursuit of sustainable H_2_ production technologies.

## Results and Discussion

### Morphology and heterostructure construction

**Fig. 1:**
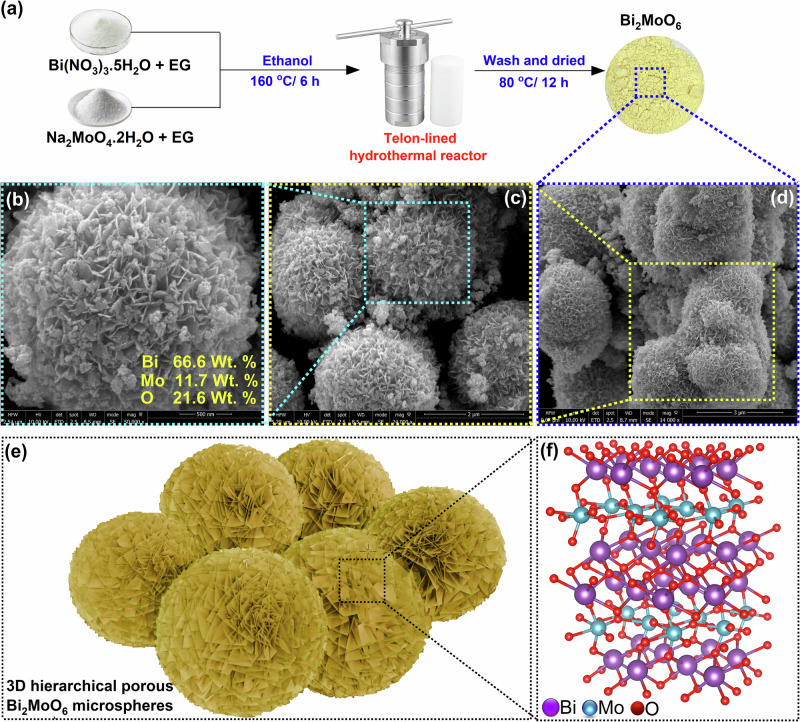
Morphology and structural model of 3D hierarchical Bi_2_MoO_6_ microspheres. **a** Schematic diagram of the experimental process for synthesizing 3D hierarchical Bi_2_MoO_6_ microspheres. **b**–**d** SEM images of Bi_2_MoO_6_ microspheres. **e** isometric projections for 3D hierarchical Bi_2_MoO_6_ microspheres. **f** Atomic structure of Bi_2_MoO_6_.

Figure 1a–f schematically illustrates the synthesis process for the formation of 3D hierarchical Bi_2_MoO_6_ microspheres. The solvothermal synthesis yielded a yellow-colored Bi_2_MoO_6_ powder, as depicted in Fig. 1a. SEM images (Fig. 1b–d) reveal the presence of uniform microspheres with an average diameter of approximately 2 ± 0.13 μm. High-magnification SEM analysis (Fig. 1b) confirms a porous 3D hierarchical structure of Bi_2_MoO_6_ composed of interconnected irregular nanosheets. Simulated molecular models of Bi_2_MoO_6_, displayed in isometric projections in Fig. 1e and 1f, provide insight into the crystal architecture. Elemental mapping by EDS, shown in Fig. S1 of the electronic supplementary information (ESI), confirms the existence of Bi, Mo, and O in the synthesized Bi_2_MoO_6_ microspheres.

**Fig. 2:**
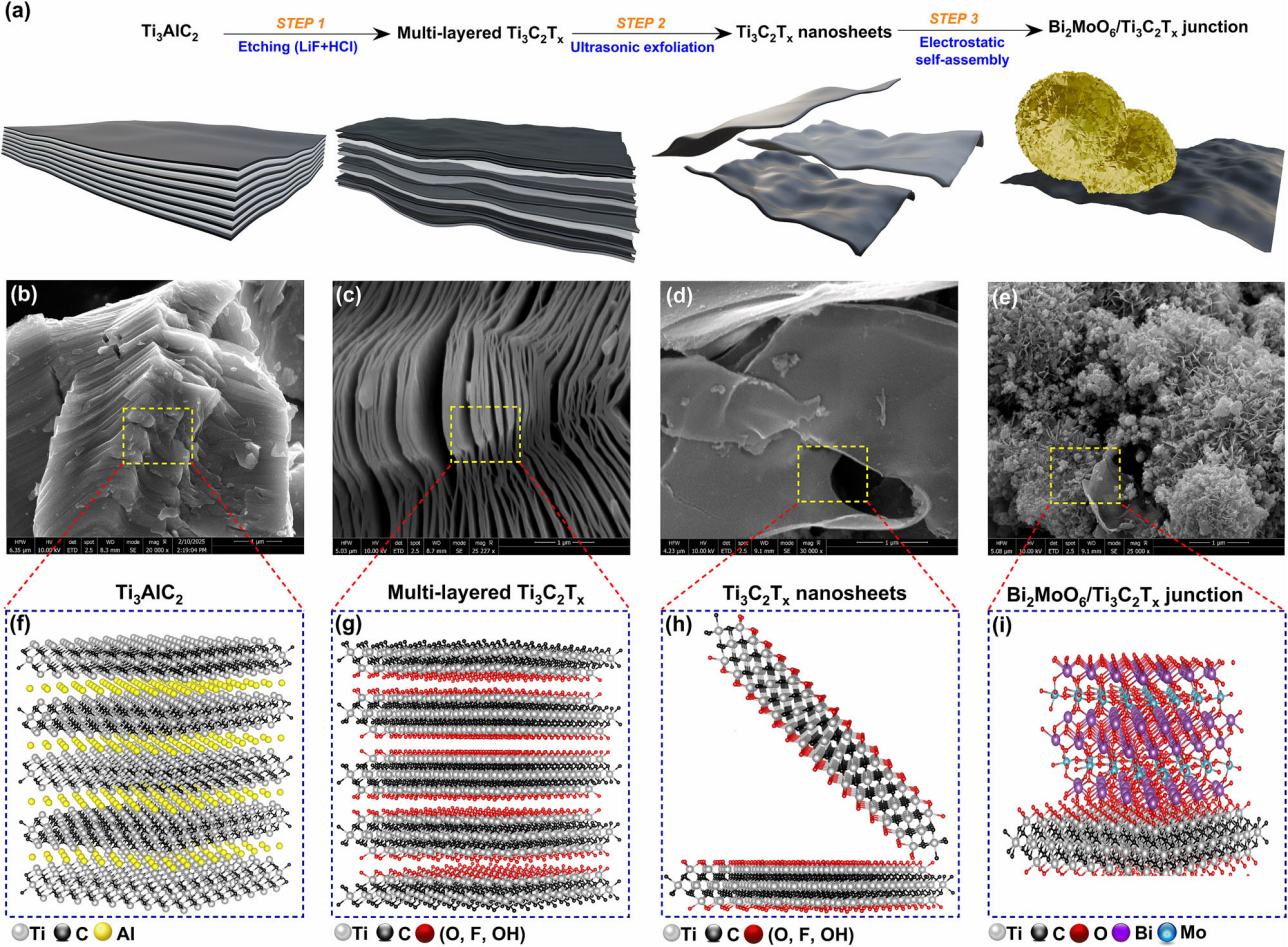
Construction of the Bi_2_MoO_6_/Ti_3_C_2_T_x_ heterostructure and morphological evolution of Ti_3_C_2_T_x_. **a** Schematic diagram of the experimental process for synthesizing Bi_2_MoO_6_/Ti_3_C_2_T_x_ composites. SEM images of **b** Ti_3_AlC_2_ MAX phase, **c** multilayered Ti_3_C_2_T_x_, **d** nanolayered Ti_3_C_2_T_x_ and **e** Bi_2_MoO_6_/Ti_3_C_2_T_x_ composites. Simulated atomic models of **f** Ti_3_AlC_2_ MAX phase, **g** multilayered Ti_3_C_2_T_x_, **h** nanolayered Ti_3_C_2_T_x_ and **i** Bi_2_MoO_6_/Ti_3_C_2_T_x_ heterojunction.

Fig. 2a presents the synthesis pathway of nanolayered Ti_3_C_2_T_x_ and the construction of Bi_2_MoO_6_/Ti_3_C_2_T_x_ heterostructure. Initially, the Al atomic layers were selectively removed from the bulk Ti_3_AlC_2_ MAX phase via in situ generated HF, producing multilayered Ti_3_C_2_T_x_ with a characteristic accordion-like 2D morphology (Fig. 2c), in contrast to the compact structure of the pristine MAX phase (Fig. 2b). The successful etching process exposes terminal surface groups such as $-{OH}$ and $-F$, attributed to the high reactivity of surface Ti atoms (Fig. 2g, 2h), as further validated by EDS analysis (Fig, S2a, S2b, ESI). Due to its hydrophilic nature, the multilayered Ti_3_C_2_T_x_ can be effectively exfoliated into 2D ultrathin Ti_3_C_2_T_x_ nanolayered sheets through ultrasonic treatment, as seen in Fig. 2d. As depicted in Fig. S2a, the multilayered Ti_3_C_2_T_x_ derived from HF etching of Ti_3_AlC_2_ displays a characteristic loose, accordion-like morphology. This structural transformation occurs as the Al layers are selectively removed and the surface is terminated with functional groups such as $-F$, $-O$, and $-{OH}$. In contrast, Fig. S2b highlights the formation of Ti_3_C_2_T_x_ nanosheets, which exhibit significantly larger lateral dimensions, enhanced flexibility, and superior mechanical robustness. These features collectively contribute to a greater exposure of electrochemically active sites. The nanolayered architecture of Ti_3_C_2_T_x_ is achieved through successive ultrasonic exfoliation steps, which overcome the weak interlayer van der Waals interactions and facilitate effective delamination.

In the subsequent step, Bi_2_MoO_6_ microspheres were integrated with the exfoliated Ti_3_C_2_T_x_ matrix via an electrostatic self-assembly approach. As shown in Fig. 2e, the resulting Bi_2_MoO_6_/Ti_3_C_2_T_x_ heterostructure preserves the 2D morphology of Ti_3_C_2_T_x_ while exhibiting a uniform distribution of Bi_2_MoO_6_ microspheres anchored to the surface without compromising the intrinsic 3D morphology of Bi_2_MoO_6_. EDS mapping (Fig. S2c, ESI) further confirms the successful formation of the heterojunction through the clear detection of Bi and Mo signals in the Bi_2_MoO_6_/Ti_3_C_2_T_x_ heterostructure. Zeta potential measurements (Fig. S3) provided direct evidence that the assembly of Bi_2_MoO_6_ microspheres with Ti_3_C_2_T_x_ MXene nanosheets is predominantly governed by electrostatic interactions. At neutral pH, pristine Bi_2_MoO_6_ exhibits a positive surface charge, whereas Ti_3_C_2_T_x_ displays a strongly negative potential. When combined, the heterostructure shows intermediate negative values that become progressively more negative with increasing MXene loading, consistent with charge compensation at the interface. Importantly, the addition of high concentrations of urea, a well-known hydrogen-bond disruptor, induced negligible variation in the zeta potential, ruling out hydrogen-bonding as the dominant interaction. Simulated atomic 3D models of both multilayered and nanolayered Ti_3_C_2_T_x_, as well as the Bi_2_MoO_6_/Ti_3_C_2_T_x_ heterojunction, are provided in isometric form in Figs. 2f–i, S4, S5 (ESI), offering a visual understanding of the heterostructure’s architecture. AFM was employed to assess the thickness of the nanolayered Ti_3_C_2_T_X_ sheets. The AFM topographic image, along with the corresponding lateral height profile, is shown in Fig. S6. The measurements indicate an average thickness of approximately ~nm, consistent with the characteristic few-layered structure of Ti_3_C_2_T_X_, thereby confirming its successful exfoliation into nanolayered sheets. Figure S7 shows the TEM and HRTEM of Bi_2_MoO_6_, multilayered and nanolayered Ti_3_C_2_T_x_. TEM images of Bi_2_MoO_6_ (Figs. S7a, S7b) show good crystallinity, and the corresponding HRTEM image (Fig. S7c) shows the lattice fringes with spacings of 0.325, 0.276, and 0.202 nm are ascribed to the (140), (200), and (080) planes of Bi_2_MoO_6_.^30^ TEM and HRTEM images of multilayered Ti_3_C_2_T_X_ (Fig. S7(d–g)) and nanolayered Ti_3_C_2_T_X_ (Fig. S7(h–k)) further confirm that multilayered structure of Ti_3_C_2_T_x_ was exfoliated into 2D Ti_3_C_2_T_x_ nanosheets through ultrasonic treatment.

**Fig. 3:**
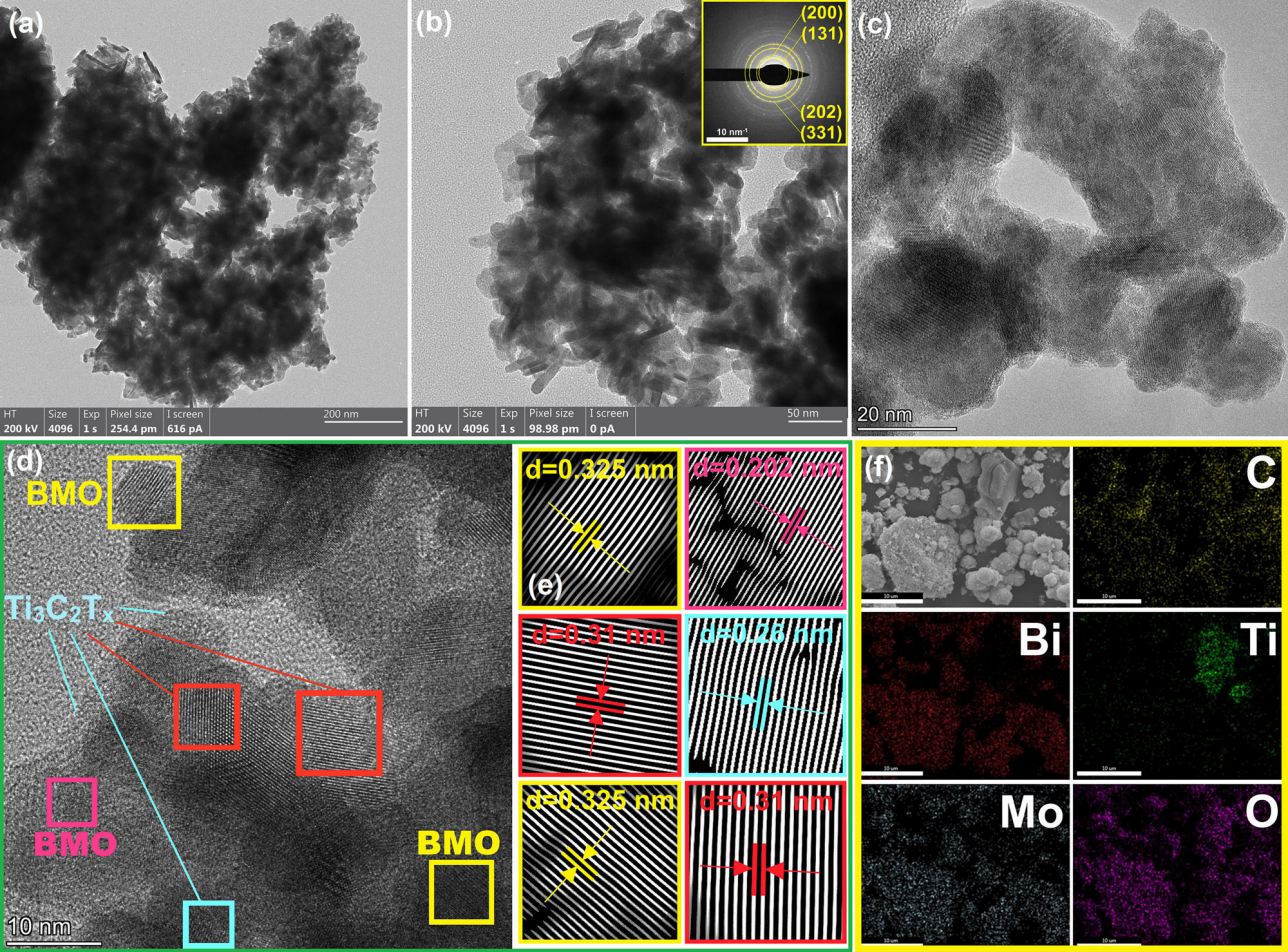
Interfacial microstructure of the Bi_2_MoO_6_/Ti_3_C_2_T_x_ heterojunction. **a**, **b** TEM images, **c**–**d** HRTEM images, **e** inverse fast Fourier transform (FFT) interfacial edges. **f** EDS mapping of Bi_2_MoO_6_/Ti_3_C_2_T_x_ heterojunction (BMT-2.5%).

Figure 3 shows the TEM and HRTEM images of Bi_2_MoO_6_/Ti_3_C_2_T_x_ heterostructure. The interfacial edge between Bi_2_MoO_6_ and Ti_3_C_2_T_x_ was clearly visualized using TEM and HRTEM, as shown in Fig. 3d. Distinct lattice fringes with spacings of 0.325 nm and 0.202 nm were observed for Bi_2_MoO_6_, while values of 0.31 nm and 0.26 nm corresponded to Ti_3_C_2_T_x_, in agreement with those of the pristine Bi_2_MoO_6_ (Fig. S7c) and Ti_3_C_2_T_x_ nanosheets (Fig. S7k), as well as previously reported data.^31,32^ Furthermore, elemental mapping via EDS (Fig. 3e) confirms the uniform distribution of Ti, C, Bi, Mo, and O throughout the Bi_2_MoO_6_/Ti_3_C_2_T_x_ heterostructure. To gain further insights into the interfacial architecture, HAADF-STEM analysis was performed (Fig. S8). The Z-contrast images clearly distinguish the heavier Bi-containing Bi_2_MoO_6_ domains from the lighter Ti-based MXene sheets, confirming the intimate integration of the two phases. Well-defined lattice fringes were observed across the junction region (as evident through TEM and HRTEM analysis), indicating coherent contact without detectable voids or interfacial gaps. The uniform contrast distribution further supports the homogeneous dispersion of Bi_2_MoO_6_ nanostructures on Ti_3_C_2_T_x_. Such tightly coupled interfaces are expected to facilitate directional charge migration across the heterostructure and play a pivotal role in suppressing charge recombination, consistent with the enhanced catalytic performance. These findings collectively validate the successful synthesis and structural integration of the Bi_2_MoO_6_/Ti_3_C_2_T_x_ heterojunction.

### Crystal structure and interfacial chemical coupling

**Fig. 4:**
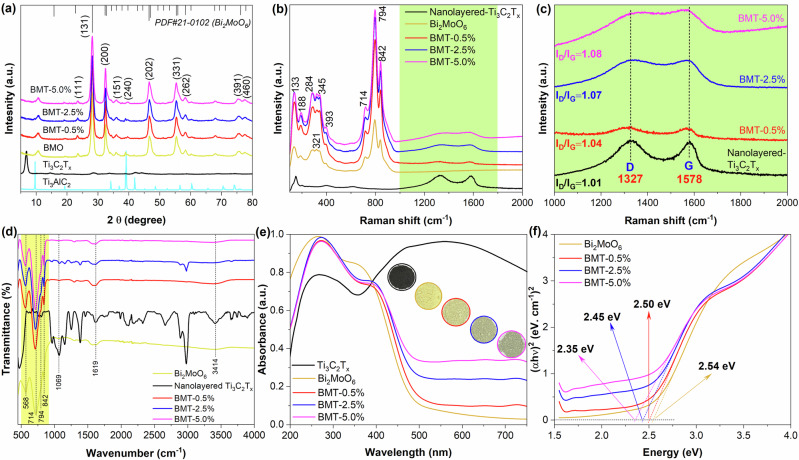
Crystal structure, vibrational properties, and optical characteristics of the Bi_2_MoO_6_/Ti_3_C_2_T_x_ composites. **a** XRD patterns of Ti_3_AlC_2_, Ti_3_C_2_T_x_, Bi_2_MoO_6_, and Bi_2_MoO_6_/Ti_3_C_2_T_x_ composites, **b** Raman spectra of Ti_3_C_2_T_x_, Bi_2_MoO_6_, and Bi_2_MoO_6_/Ti_3_C_2_T_x_ composites, **c** Enlarged Raman spectra of Ti_3_C_2_T_x_ and Bi_2_MoO_6_/Ti_3_C_2_T_x_ composites, **d** FTIR spectra of Ti_3_C_2_T_x_, Bi_2_MoO_6_, and Bi_2_MoO_6_/Ti_3_C_2_T_x_ composites, **e** UV-vis DRS, and **f** Tauc plot of Bi_2_MoO_6_, and Bi_2_MoO_6_/Ti_3_C_2_T_x_ composites.

The powder XRD analysis was employed to investigate the stacking configurations and crystalline phases of Ti_3_AlC_2_, Ti_3_C_2_T_x_, and the Bi_2_MoO_6_/Ti_3_C_2_T_x_ heterostructures, as depicted in Fig. 4a. The pristine Ti_3_AlC_2_ reveals sharp and well-defined diffraction peaks at 9.5°, 19.1°, 33.9°, 36.2°, 38.9°, 41.7°, 44.9°, 48.5°, 56.3°, 60.1°, and 65.2°, which correspond to the (002), (004), (101), (102), (104), (105), (106), (107), (109), (110), and (1011) planes of the MAX phase Ti_3_AlC_2_ (ICDD PDF No. 52-0875)^33^, respectively. Upon HF etching, these characteristic peaks largely disappear, except for the (002) reflection at 9.5°, which shifts to a lower angle, indicative of an expanded *c*-lattice parameter (~2.6 Å).^33,34^ This shift confirms the effective removal of Al atoms and the formation of nanolayered Ti_3_C_2_T_x_ with increased interlayer *d* spacing, characteristic of delaminated structures. The broadening and attenuation of the (002) peak further suggest a reduction in stacking order, consistent with few-layered or amorphous-like in-plane structures. Notably, the absence of peaks corresponding to anatase TiO_2_ and residual Ti_3_AlC_2_ phases confirms the high phase purity and complete transformation to Ti_3_C_2_T_x_. In parallel, the XRD profile of Bi_2_MoO_6_ reveals the orthorhombic crystal structure (space group *Pna*_*2*_*₁*, ICDD PDF No. 21-0102), with lattice parameters *a* = 5.502 Å, *b* = 16.213 Å, and *c* = 5.483 Å, and no secondary crystalline phases were detected.^31,35^ The successful deposition of Bi_2_MoO_6_ onto nanolayered Ti_3_C_2_T_x_ surfaces is evidenced by the appearance of diffraction peaks attributable to orthorhombic Bi_2_MoO_6_ in all heterojunction samples. However, Ti_3_C_2_T_x_-related peaks are scarcely visible in the Bi_2_MoO_6_/Ti_3_C_2_T_x_ composites, likely due to their low loading and the extensive coverage by Bi_2_MoO_6_ microspheres, as supported by SEM and TEM analyses.

Raman spectroscopy was employed to gain deeper insights into the structural evolution of the materials. As illustrated in Fig. S9, the Raman spectra of Ti_3_AlC_2_ and nanolayered Ti_3_C_2_T_x_ were compared to elucidate changes induced by etching and exfoliation. For Ti_3_AlC_2_, the characteristic peaks *ω*_*1*_ and *ω*_*2*_ at 145 cm⁻¹ and 266 cm⁻¹ are attributed to ${Ti}-{Al}$ vibrational modes, whereas *ω*_*3*_ and *ω*_*4*_ at 413 cm⁻¹ and 602 cm⁻¹ correspond to ${Ti}-C$ bond vibrations.^34,36^ In contrast, the Raman spectrum of nanolayered Ti_3_C_2_T_x_ shows a complete suppression of the ω_1_ and ω_2_ modes, along with significant broadening and intensity reduction of the ω_3_ and ω_4_ peaks, which is indicative of weakened long-range order and increased structural disorder following Al removal. A prominent sharp peak at 149 cm⁻¹, characteristic of resonant Ti_3_C_2_T_x_, further validates successful MXene formation, consistent with previous literature.^36,37^ Fig. 4b presents the Raman spectrum of Bi_2_MoO_6_, where modes at approximately 133 and 188 cm⁻¹ are associated with lattice vibrations of the (Bi_2_O_2_)^2+^ layers.^38^ Additional bands at 284, 321, 345, 393, 714, 794, and 842 cm⁻¹ are assigned to the symmetric and asymmetric stretching modes of the MoO_6_ octahedra, particularly involving apical oxygen motion relative to the (Bi_2_O_2_)^2+^ framework.^39,40^

Furthermore, magnified Raman spectra (Fig. 4c) reveal distinct D and G bands at 1327 and 1578 cm⁻¹, respectively, which are characteristic of *sp*^*2*^-hybridized carbon.^41^ These bands are observed exclusively in Ti_3_C_2_T_x_ and Bi_2_MoO_6_/Ti_3_C_2_T_x_ composites. Notably, the Bi_2_MoO_6_/Ti_3_C_2_T_x_ heterostructures exhibit a slight downshift in the G band along with an increased I_D_/I_G_ ratio, suggesting enhanced defect density and changes in the in-plane vibrations of *sp*^*2*^ carbon atoms, indicative of effective interfacial charge transfer between Bi_2_MoO_6_ and Ti_3_C_2_T_x_.^41^ Overall, the Raman findings further reinforce the structural transformation of Ti_3_AlC_2_ into nanolayered Ti_3_C_2_T_x_ through selective etching and ultrasonic exfoliation, followed by interfacial Bi_2_MoO_6_/Ti_3_C_2_T_x_ heterojunction, which is in agreement with the powder XRD results showing loss of crystallinity and increased disorder.

Figure 4d presents the FTIR spectra of pristine Bi_2_MoO_6_, Ti_3_C_2_T_x_, and Bi_2_MoO_6_/Ti_3_C_2_T_x_ composites. In the spectrum of pristine Bi_2_MoO_6_, characteristic bands at 568 and 714 cm⁻¹ are attributed to the bending vibrations of MoO_6_ octahedra and the asymmetric stretching of ${Mo}-O$ bonds, respectively.^42^ Additional absorption peaks at 794 and 842 cm⁻¹ correspond to the symmetric and asymmetric ${Mo}-O$ stretching modes within the MoO_6_ framework.^43^ A weaker band at approximately 454 cm⁻¹ is ascribed to the stretching and bending vibrations of BiO_6_ octahedral units.^44^ For the Ti_3_C_2_T_x_ spectrum, distinct peaks are observed at 3414 cm⁻¹ and 1619 cm⁻¹, which are assigned to $O-H$ stretching and bending vibrations, indicating surface hydroxylation.^45^ The bands at 1069 cm⁻¹ and 475 cm⁻¹ correspond to $C-O-C$ and ${Ti}-C$ vibrational modes, respectively, ascribed to surface terminations and structural features of MXene sheets.^46^ In the Bi_2_MoO_6_/Ti_3_C_2_T_x_ composites, slight shifts in the ${Bi}-O$ and ${Mo}-O$ vibrational bands suggest strong interfacial interactions between the two components. Moreover, the broadening of peaks near ~3400 cm⁻¹ and ~1630 cm⁻¹ becomes more evident with increasing Ti_3_C_2_T_x_ content, which may be attributed to enhanced surface hydroxylation or increased water adsorption.^47^ Collectively, the FTIR spectra confirm that the essential vibrational features of both Bi_2_MoO_6_ and Ti_3_C_2_T_x_ are retained in the composite structure, while spectral shifts and band broadening provide further evidence of successful heterojunction formation and interfacial coupling.

### Electronic Structure and Surface Chemical States

A well-matched energy band alignment is critical for facilitating piezocatalytic processes, and it can be effectively evaluated by integrating UV-vis DRS and XPS valence band analysis. As shown in Fig. 4e, pristine Bi_2_MoO_6_ exhibits a distinct absorption edge at approximately 488 nm, while Ti_3_C_2_T_x_ absorbs light across the entire examined spectral range, which is attributed to its near-zero band gap. Upon incorporation of Ti_3_C_2_T_x_, a minor redshift in the absorption edge is observed, potentially attributed to interfacial interactions or edge-state modifications induced by hybridization between Bi_2_MoO_6_ and Ti_3_C_2_T_x_.^48^ Moreover, due to the submetallic nature of Ti_3_C_2_T_x_, the composites demonstrate enhanced absorption intensity in the $420-750$ nm range, which increases progressively with higher Ti_3_C_2_T_x_ content as evidenced by the visually darker coloration of the samples shown in the inset of Fig. 4e. The optical band gaps of the synthesized materials were estimated via Tauc plots derived from the Kubelka-Munk function, αhν = A(*hν*–*E*_g_)^n/2^, where α is the absorption coefficient, *h* is Planck’s constant, *ν* the photon frequency, *E*_g_ the bandgap, and *A* is a proportionality constant. For Bi_2_MoO_6_, which is a direct bandgap semiconductor, the exponent n is taken as 1.^49^ As depicted in Fig. 4f, the bandgap values were extracted to be 2.54 eV and 2.45 eV for Bi_2_MoO_6_ and BMT-2.5% composite, respectively. To further elucidate the electronic structure, XPS valence band measurements (Fig. S10) reveal the valence band maximum (*E*_VB_) of Bi_2_MoO_6_ to be approximately 1.59 eV. This value was converted to the standard hydrogen electrode (SHE) scale using the equation: *E*_VB–NHE_ = Φ+*E*_VB(XPS)_–4.4, where Φ (the analyzer work function) is 4.50 eV, yielding a *E*_VB_ position of 1.69 V *vs*. NHE. Applying the relation *E*_CB_ = *E*_VB_ – *E*_g_,^50^ the conduction band edge (*E*_CB_) position of Bi_2_MoO_6_ is calculated to be $-0.85$ V. This favorable band structure supports efficient piezocatalytic reduction, particularly the generation of H_2_ via proton reduction. Furthermore, the integration of highly conductive Ti_3_C_2_T_x_ introduces a highly conductive pathway that promotes the rapid migration of photoexcited electrons from the *E*_CB_ of Bi_2_MoO_6_ to Ti_3_C_2_T_x_, thereby enhancing charge separation and boosting overall catalytic performance.

**Fig. 5:**
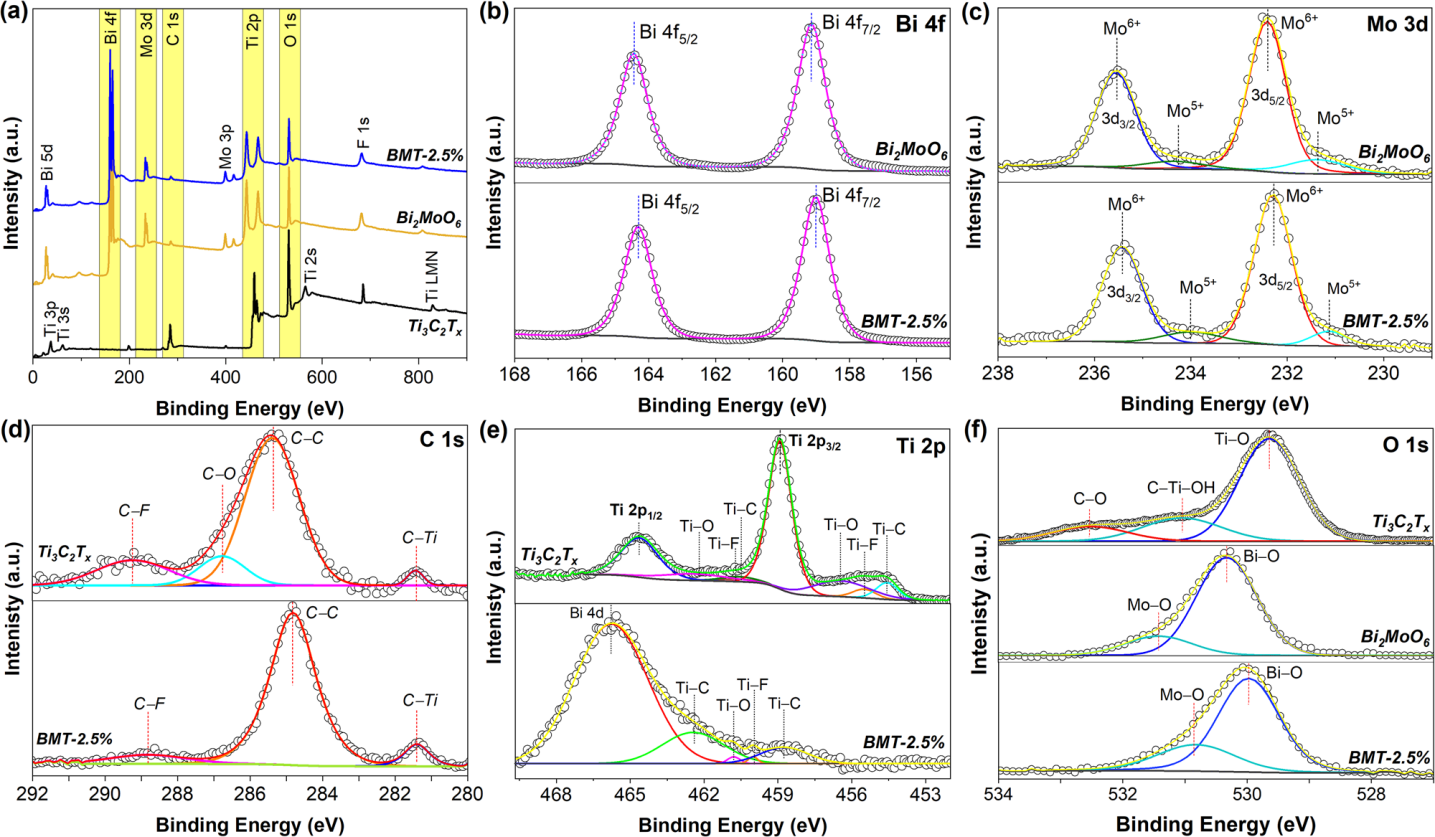
Surface chemical states and interfacial electronic interactions in the Bi_2_MoO_6_/Ti_3_C_2_T_x_ heterostructure. **a** XPS survey of Ti_3_C_2_T_x_, Bi_2_MoO_6_, and Bi_2_MoO_6_/Ti_3_C_2_T_x_ (BMT-2.5%) composite, **b** High-resolution Bi 4 f spectra in Bi_2_MoO_6_ and BMT-2.5%, **c** High-resolution Mo 3 d spectra in Bi_2_MoO_6_ and BMT-2.5%, **d** High-resolution Bi 4 f spectra in Ti_3_C_2_T_x_ and BMT-2.5%, **e** High-resolution Ti 2p spectra in Ti_3_C_2_T_x_ and BMT-2.5%, and **f** High-resolution O 1 s spectra in Ti_3_C_2_T_x_, Bi_2_MoO_6_, and BMT-2.5%.

To elucidate the surface chemical composition and valence states of as-prepared materials, XPS was employed. Figure 5a shows the survey XPS spectrum of Ti_3_C_2_T_x_, Bi_2_MoO_6_, and Bi_2_MoO_6_/Ti_3_C_2_T_x_ (BMT-2.5%) composites. The XPS survey spectra (Fig. 5a) confirm the presence of C 1s, O 1 s, Ti 2p, Bi 4f, and Mo 3d signals in BMT-2.5%, consistent with elemental mapping and EDS results. High-resolution spectra of Bi 4 f and Mo 3 d (Fig. 5b, c) reveal no significant shift in the oxidation states compared to pristine Bi_2_MoO_6_, with Bi 4f_7/2_ and Bi 4f_5/2_ peaks at 159.0 and 164.3 eV, characteristic of Bi^3+^, and Mo 3d_5/2_ and Mo 3d_3/2_ at 232.3 and 235.4 eV, indicating Mo^6+^ in Bi_2_MoO_6_ microspheres.^16,51^ However, a slight positive shift in binding energies for Bi and Mo in the BMT-2.5% composite suggests enhanced electron density and strong interfacial electronic interactions between Bi_2_MoO_6_ and Ti_3_C_2_T_x_, indicative of directional charge transfer pathways. In Fig. 5d, the C 1 s spectrum of pristine Ti_3_C_2_T_x_ displays four main components: ${Ti}-C$ at 281.4 eV, $C-{C}$ (graphitic carbon) at 284.8 eV, $C-O$ from surface adsorbates at 286.7 eV, and a $C-F$ peak at 289.0 eV, originating from terminal groups on MXene surfaces.^32,52^ In the BMT-2.5% composite, the C 1 s spectrum similarly exhibits peaks at 281.4, 284.8, and 288.8 eV, attributed to ${Ti}-C,{C}-C$, and $C-F$ bonds, respectively, confirming the structural integrity of Ti_3_C_2_T_x_ within the heterostructure. High-resolution Ti 2p spectra (Fig. 5e) further reveal distinct features for ${Ti}-C$ (2p_3/2_ at 458.8 eV and 2p_1/2_ at 464.6 eV), Ti^3+^ oxide (456.4 and 460.4 eV), ${Ti}-O$ (456.4 and 462.1 eV), and ${Ti}-F$ (455.5 and 460.6 eV) in pristine nanolayered Ti_3_C_2_T_x_.^53,54^ Notably, the ${Ti}-C$ components at 281.4 eV and 458.8 eV in the C 1 s and Ti 2p spectrum, respectively, suggests the presence of MXene-derived ${Ti}-C$ bonding.^53^ Interestingly, the low-valence Ti 2p component is significantly diminished or absent in the BMT-2.5% composite (Fig. 5e), indicating its possible consumption during the composite formation, possibly due to interfacial redox reactions.^55^ Additionally, the Ti 2p peaks in BMT-2.5% exhibit a slight negative shift relative to pristine Ti_3_C_2_T_x_, suggesting interfacial electron transfer from Bi_2_MoO_6_ to Ti_3_C_2_T_x_, which facilitates effective charge carrier separation. Figure 5f illustrates the high-resolution O 1 s spectra. For Ti_3_C_2_T_x_, three components are evident at 529.6 eV (lattice oxygen), 531.0 eV ($C-{Ti}-{OH}$), and 532.5 eV ($C-O$).^53^ In contrast, Bi_2_MoO_6_ displays peaks at 530.3 and 531.4 eV, corresponding to ${Bi}-O$ and ${Mo}-{O}$ bonds,^15,31^ respectively. In the BMT-2.5% composite, these peaks shift to lower binding energies, further supporting interfacial electronic redistribution. From a structural standpoint, the presence of surface O-terminal groups on Ti_3_C_2_T_x_ disrupts inversion symmetry in the $x-{y}$ plane of monolayer MXene, a desirable feature for enhancing piezocatalytic activity.^29^ These functional groups also serve as active sites for electrostatic interaction with Bi_2_MoO_6_, leading to intimate interfacial contact and local atomic coordination. Such interactions enable the formation of a robust heterojunction with efficient charge transport channels and reduced migration distances between Bi_2_MoO_6_ and Ti_3_C_2_T_x_. Electrons excited in Bi_2_MoO_6_ are transferred to the more electronegative Ti_3_C_2_T_x_, where they accumulate at surface-active sites, modulating the interfacial electronic structure via a Schottky junction. This promotes favorable H_2_ adsorption thermodynamics, offering high-performance active sites for the H_2_ evolution reaction. Therefore, the formation of a Bi_2_MoO_6_/Ti_3_C_2_T_x_ Schottky junction could significantly enhance H_2_ evolution efficiency via improved charge separation and transport across the heterojunction.

### Piezocatalytic hydrogen evolution performance

**Fig. 6:**
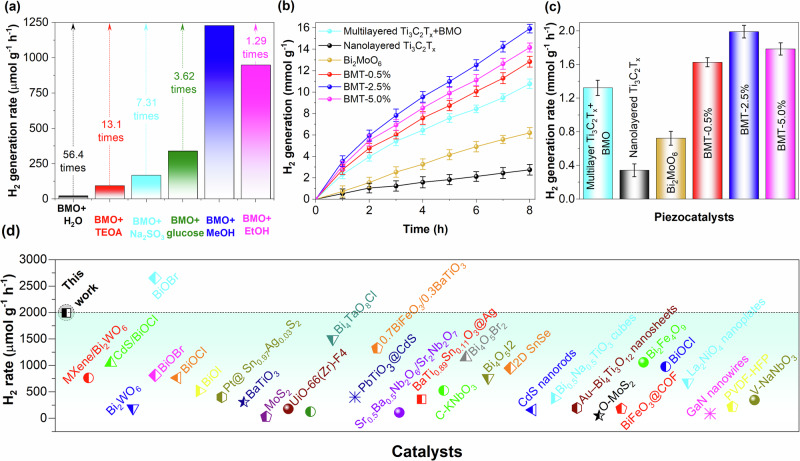
Piezocatalytic hydrogen evolution performance of the synthesized catalysts. **a** Hydrogen evolution experiments under different reaction conditions (BMO in H_2_O, TEOA, Na_2_SO_3_, glucose, MeOH, and EtOH) [BMO=Bi_2_MoO_6_]. **b** Time-yield profiles of hydrogen (${mmol}{g}^{-1}$) and (**c**) comparison of hydrogen evolution rates of all piezocatalysts (${mmol}{g}^{-1}\,{h}^{-1}$). **d** Comparison of piezocatalytic hydrogen evolution performance over BMT-2.5% to the other typical reported piezocatalysts.

To assess the piezocatalytic performance of the synthesized catalysts, we conducted H_2_ evolution experiments under ultrasonic vibration. Initially, the piezocatalytic H_2_ generation ability of pristine Bi_2_MoO_6_ was examined under various conditions. As shown in Fig. 6a, negligible H_2_ evolution was observed in pure water, which can be attributed to rapid charge carrier recombination, thereby limiting the availability of photogenerated electrons for proton reduction.^32,56^ Importantly, the introduction of a sacrificial agent was found to play a pivotal role in enhancing the piezocatalytic H_2_ evolution efficiency. These agents function by scavenging photogenerated holes, thereby suppressing recombination and prolonging electron lifetimes for effective H^+^ reduction.^57^ To systematically evaluate the impact of different sacrificial agents, H_2_ evolution was tested using TEOA, Na_2_SO_3_, glucose, MeOH, and EtOH as sacrificial agents. Among these, MeOH yielded the highest enhancement in H_2_ production over 5 hours, exhibiting rate increases by factors of 56.4, 13.1, 7.31, 3.62, and 1.29 compared to H_2_O, TEOA, Na_2_SO_3_, glucose, and EtOH, respectively. A maximum H_2_ evolution rate of 1.228 ${mmol}{g}^{-1}{h}^{-1}$ was achieved with MeOH, outperforming all other sacrificial agents. A MeOH/H_2_O mixed solvent (1:4, v/v) was employed to maintain sufficient proton supply and stable cavitation in water, while a moderate methanol fraction efficiently scavenges piezo-generated holes and suppresses charge recombination. Consequently, MeOH was selected as the sacrificial agent for subsequent experiments, in alignment with previous literature reports.^58–61^ It should be emphasized that the sacrificial agents primarily function as hole scavengers to suppress hole-electron recombination and prolong electron lifetime for H^+^ reduction. Under ultrasonic excitation, these molecules may undergo oxidation via piezo-generated holes and reactive oxygen species on the catalyst surface (carrier-mediated oxidation), rather than being directly decomposed by the piezopotential itself.^62,63^

Figure 6b presents the time-dependent hydrogen evolution profiles for the as-synthesized piezocatalysts. The optimized BMT-2.5% sample exhibits a significantly enhanced H_2_ yield of 15.93 ${mmol}{g}^{-1}$ over an 8 h ultrasonic reaction, outperforming pristine Bi_2_MoO_6_ (6.19 ${mmol}{g}^{-1}$), pristine Ti_3_C_2_T_x_ (2.75 ${mmol}{g}^{-1}$), BMT-0.25% (12.84 ${mmol}{g}^{-1}$), and BMT-5.0% (14.17 ${mmol}{g}^{-1}$). Although bulk Ti_3_C_2_T_x_ is not a classical piezoelectric, the asymmetric distribution of surface terminations (T_x_ = O/OH/F) can break local inversion symmetry and enable a measurable polarization/charge response under ultrasonic deformation, as supported by PFM results. Once generated, these carriers can be efficiently consumed for H^+^ reduction because Ti_3_C_2_T_x_ provides high conductivity and favorable H_2_ evolution reaction surface energetics; therefore, a modest yet reproducible H_2_ evolution is observed for pristine Ti_3_C_2_T_x_ under vibration.^14,32^ Notably, when an equivalent amount of multilayer Ti_3_C_2_T_x_ is used with Bi_2_MoO_6_, the resulting composite displays a comparatively lower H_2_ yield of 10.77 ${mmol}{g}^{-1}$. This suggests that the superior performance of BMT-2.5% is attributed to the formation of an interfacial Schottky junction between nanolayered Ti_3_C_2_T_x_ and Bi_2_MoO_6_, as illustrated in Fig. 2 and 3. This interfacial Schottky junction effectively promotes charge separation and directional electron transfer. However, a further increase in Ti_3_C_2_T_x_ loading to 5.0% leads to a reduction in piezocatalytic activity. This decline is likely to be due to excess Ti_3_C_2_T_x_ serving as recombination centers, which hinder charge transport and reduce the availability of free carriers for the redox reaction.^64,65^ Complementary to these findings, the corresponding H_2_ evolution rates (Fig. 6c) for Bi_2_MoO_6_, nanolayered Ti_3_C_2_T_x_, multilayered Bi_2_MoO_6_/Ti_3_C_2_T_x_, BMT-0.5%, BMT-2.5%, and BMT-5.0% are 0.72 ± 0.082, 0.34 ± 0.076, 1.32 ± 0.091, 1.62 ± 0.057, 1.99 ± 0.075, and 1.78 ± 0.072 ${mmol}{g}^{-1}\,{h}^{-1}$, respectively. Remarkably, BMT-2.5% achieves the highest rate, representing a 2.74-fold and 5.79-fold enhancement over pristine Bi_2_MoO_6_ and Ti_3_C_2_T_x_, respectively. Remarkably, the piezocatalytic H_2_ evolution performance of BMT-2.5% surpasses that of most previously reported piezocatalysts, as illustrated in Fig. 6d and detailed in Table S1. Notably, a growing number of high-performance piezocatalysts reported are based on polar layered bismuth-containing compounds, such as BiOX (X = Cl, Br, I) and Aurivillius-type bismuth oxides (e.g., Bi_2_WO_6_). These materials possess intrinsically polarized layered crystal structures, often arising from alternating charged slabs and stereochemically active Bi^3+^ lone pairs, which facilitate vibration-induced polarization and efficient charge separation under mechanical excitation.^18–20^ The increasing prevalence of layered Bi-based systems among state-of-the-art piezocatalysts highlights their promise for mechanically driven H_2_ evolution and provides a strong structural rationale for further performance enhancement through interfacial engineering, as demonstrated by the Bi_2_MoO_6_/Ti_3_C_2_T_x_ heterostructure in this work. To further assess its durability, cyclic H_2_ evolution experiments were conducted (Fig. S11a, ESI). Only a marginal decline in H_2_ yield was observed after the fourth cycle, highlighting the excellent piezocatalytic stability and reusability of BMT-2.5%. The structural analyses were carried out using XRD, FTIR, and SEM to evaluate the physical and chemical integrity of BMT-2.5% after prolonged ultrasonic treatment. The powder XRD pattern (Fig. S11b) displays negligible changes compared to the fresh piezocatalyst, indicating the preservation of its crystalline structure. Similarly, FTIR spectra (Fig. S11c) reveals the retention of vibrational bands, confirming that the functional groups and chemical framework remain intact. SEM imaging (Fig. S11d) further verifies the morphological stability of the catalyst. These findings collectively demonstrate that the strategic incorporation of an optimal amount of nanolayered Ti_3_C_2_T_x_ into Bi_2_MoO_6_, along with the formation of an interfacial Schottky junction, significantly enhances piezocatalytic H_2_ evolution while maintaining structural stability and excellent cycling performance.

### Piezoelectric response and charge-transfer behavior

**Fig. 7:**
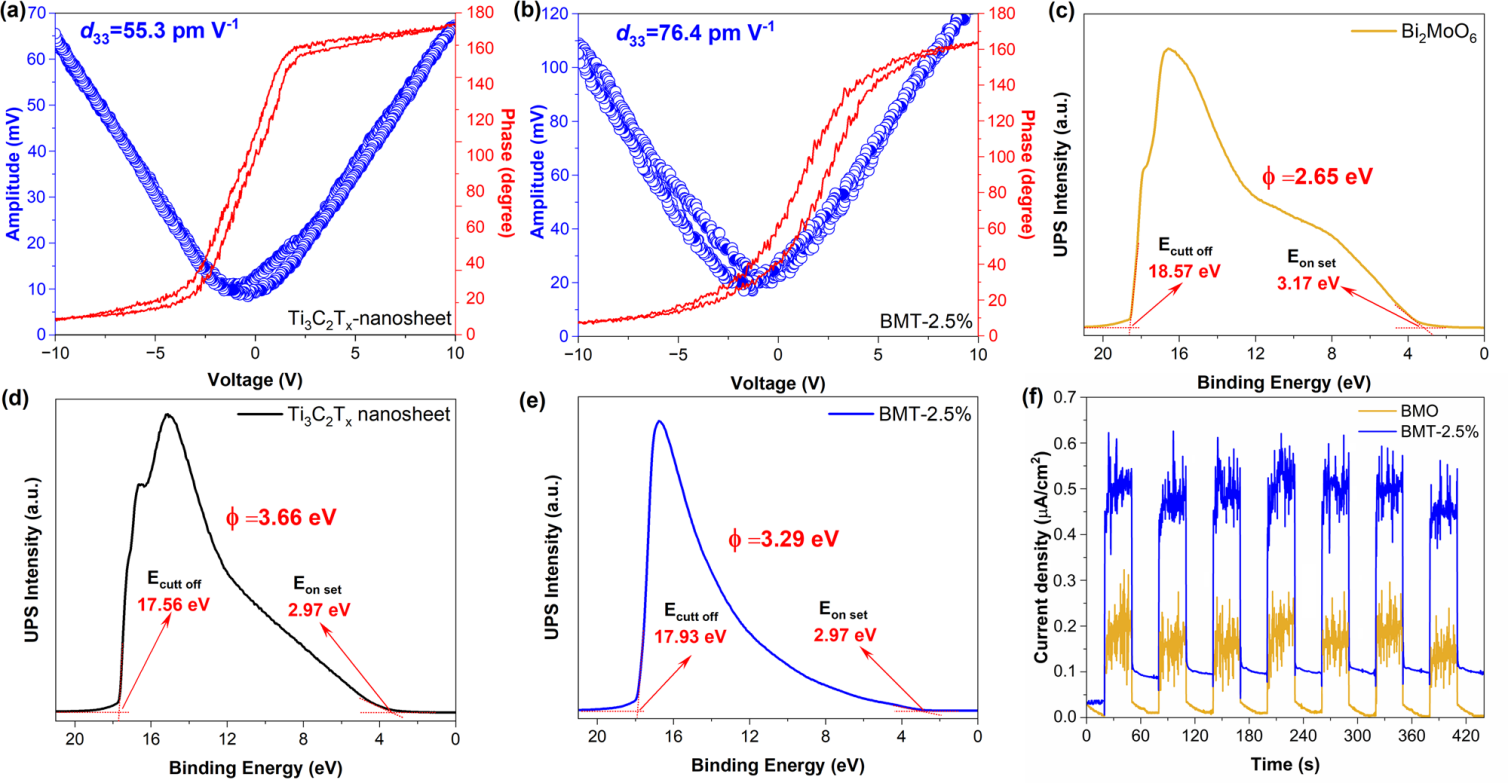
Piezoelectric response, work-function analysis, and charge-transfer behavior of the Bi_2_MoO_6_/Ti_3_C_2_T_x_ heterostructure. PFM amplitude-butterfly and phase hysteresis loop of **a** Ti_3_C_2_T_x_, and **b** Bi_2_MoO_6_/Ti_3_C_2_T_x_ (BMT-2.5%) composite. UPS spectra of **c** Bi_2_MoO_6_, **d** Ti_3_C_2_T_x_, and **e** BMT-2.5%. **f** Transient piezocurrent response *vs*. time plot of Bi_2_MoO_6_ and BMT-2.5%.

Piezoresponse force microscopy (PFM) was further employed to investigate the piezoelectric behavior of pristine Ti_3_C_2_T_x_ and the Bi_2_MoO_6_/Ti_3_C_2_T_x_ composite (BMT-2.5%). As shown in Fig. 7a, b, the phase and amplitude maps exhibit good spatial correlation, indicative of pronounced piezoelectric activity in Ti_3_C_2_T_x_ and BMT-2.5%. The piezoelectric constants (*d*_33_) were calculated based on the characteristic butterfly-shaped amplitude-voltage responses (Fig. 7a, b). Notably, the BMT-2.5% composite exhibited a significantly enhanced *d*_33_ value of 76.4 pm V^−1^, compared to 55.3 pm V^−1^ for Ti_3_C_2_T_x_. The growth of the amplitude loops into asymmetric butterfly curves suggests the presence of imprint, which is a defect likely arising from electrical imprint field.^66^ Furthermore, phase hysteresis loops revealed ∼180° switching behavior under an applied tip bias of ±10 V, confirming the occurrence of polarization reversal.^67,68^ Compared to the pristine Ti_3_C_2_T_x_, the BMT-2.5% composite exhibited more distinct and robust bipolar domain contrast, supporting the role of the Bi_2_MoO_6_ in enhancing ferroelectric domain formation and switching behavior.^69^ These findings collectively demonstrate the improved piezoelectric response and remnant polarization of BMT-2.5%, which are expected to contribute positively to its piezocatalytic performance.

To confirm the formation of a Schottky junction at the Bi_2_MoO_6_/Ti_3_C_2_T_x_ interface, UPS was employed to determine the work functions (Φ) of pristine Bi_2_MoO_6_, Ti_3_C_2_T_x_, and BMT-2.5% composite (Fig. 7c–e). The secondary electron cutoff energies (*E*_cutoff_) for Bi_2_MoO_6_, Ti_3_C_2_T_x_, and BMT-2.5% were measured to be 18.57, 17.56, and 17.93 eV, respectively, relative to a Fermi level (*E*_f_) of 0. Using the relation Φ = 21.2 − |*E*_cutoff_ − *E*_f_ | , the calculated work functions were determined as 2.65 eV for Bi_2_MoO_6_, 3.66 eV for Ti_3_C_2_T_x_, and 3.29 eV for BMT-2.5% (*vs*. vacuum level).^43,70^ The intermediate value of Φ for BMT-2.5% suggests electronic interaction between Bi_2_MoO_6_ and Ti_3_C_2_T_x_, which is indicative of balancing the Fermi level at the heterojunction interface. Consequently, electrons spontaneously transfer from Bi_2_MoO_6_ (lower Φ) to Ti_3_C_2_T_x_ (higher Φ) until equilibrium is achieved, resulting in upward energy band bending in Bi_2_MoO_6_ and the establishment of a Schottky junction. This junction plays a critical role in suppressing electron backflow, thereby promoting directional charge separation. The presence of the Schottky junction enhances interfacial charge transport, effectively reduces electron-hole recombination, and increases the density of active sites at the interface. These synergistic effects facilitate efficient and unidirectional electron transfer, ultimately boosting piezocatalytic performance.^70,71^ Under ultrasonic excitation, the piezopotential primarily enhances charge separation and directional hole migration to the catalyst surface, where the accumulated holes and associated reactive oxygen species drive the oxidation of sacrificial agents via carrier-mediated pathways, rather than through direct decomposition by the polarization field itself.

To elucidate the differences in charge separation efficiency and carrier transport mechanisms between pristine Bi_2_MoO_6_ and the BMT-2.5% Schottky junction, a combination of piezoelectrochemical measurements and spectroscopic techniques was employed, as presented in Fig. 7f and S12. The transient piezocurrent response under periodic ultrasonic vibrations (Fig. 7f) reveals that BMT-2.5% generates a significantly higher piezocurrent density compared to Bi_2_MoO_6_. This enhancement indicates improved charge separation efficiency in BMT-2.5%, attributed to the presence of the Schottky interface facilitating directional charge flow and minimizing recombination. These results are further supported by linear sweep voltammetry (LSV) and electrochemical impedance spectroscopy (EIS) analyses (Fig. S12). LSV measurements conducted in the absence of light (Fig. S12a) show that BMT-2.5% exhibits a pronounced anodic current, affirming its intrinsic *n*-type semiconducting nature. Moreover, the observed current for BMT-2.5% is significantly greater than that of pristine Bi_2_MoO_6_, highlighting the enhanced charge carrier mobility and effective separation induced by the Schottky junction. In addition, EIS Nyquist plots (Figure S12(b)) reveal a markedly smaller arc radius for BMT-2.5% in comparison to other synthesized piezocatalysts, indicating a substantial reduction in charge transfer resistance. This observation confirms that the formation of a Schottky barrier at the Bi_2_MoO_6_/Ti_3_C_2_T_x_ interface significantly facilitates interfacial charge transport and suppresses carrier recombination, thereby contributing to the superior piezocatalytic performance of BMT-2.5%. Furthermore, steady-state photoluminescence (PL) spectroscopy results, as shown in Figure S12c, demonstrates that BMT-2.5% exhibits a reduced PL intensity compared to pristine Bi_2_MoO_6_, indicating a lower electron–hole recombination rate and more efficient charge separation within the composite. These findings suggest that the superior piezocatalytic H_2_ evolution performance of the Bi_2_MoO_6_/Ti_3_C_2_T_x_ heterostructure is predominantly attributed to the efficient separation of charge carriers and the accelerated interfacial charge transfer enabled by the formation of a Schottky junction.

### DFT analysis and proposed piezocatalytic mechanism

**Fig. 8:**
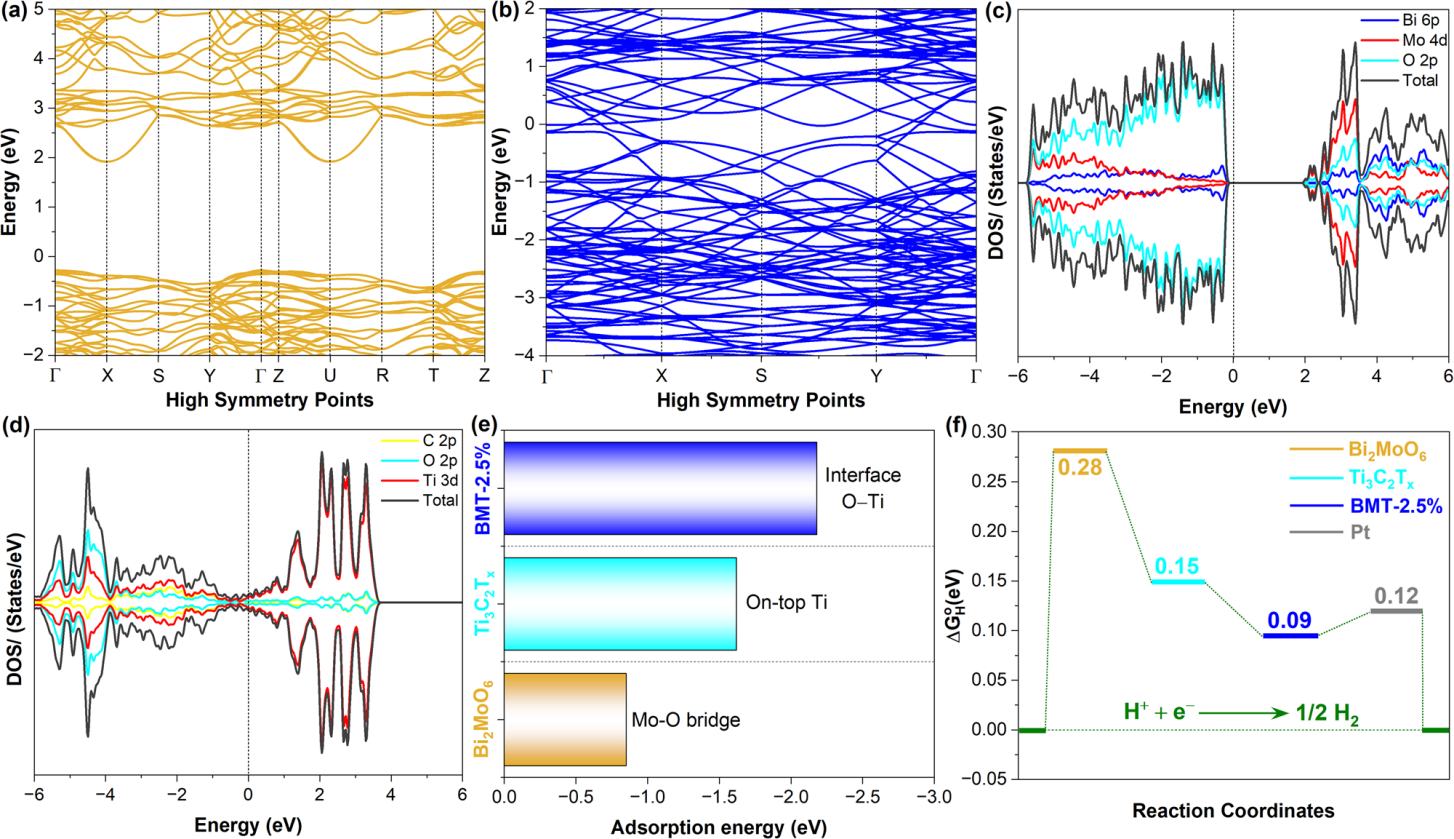
DFT analysis of the electronic structure and hydrogen evolution energetics. Band structures of **a** Bi_2_MoO_6_ and **b** Ti_3_C_2_T_x_. Calculated density of states (DOS) for **c** Bi_2_MoO_6_ and **d** Ti_3_C_2_T_x_. **e** Adsorption energy and preferred site of water on Bi_2_MoO_6_, Ti_3_C_2_T_x_, and BMT-2.5%. **f** Gibbs free energy diagram of hydrogen generation in catalysts.

We conducted density functional theory (DFT) calculations using the projected augmented wave (PAW) method to ascertain the electronic band structures, interfacial electron-transfer behavior, and adsorption properties to gain a deeper understanding of the catalytic mechanism of the Bi_2_MoO_6_/Ti_3_C_2_T_x_ Schottky junction during the piezocatalytic hydrogen evolution process. Bi_2_MoO_6_ is a direct band gap semiconductor, as shown in Fig. 8a. Its calculated band gap of 2.21 eV slightly underestimates the experimental value of 2.54 eV due to the exchange-correction functional’s limitations when probing excited states, as has been documented in previous studies.^72,73^ On the other hand, Ti_3_C_2_T_x_ energy band structures show continuous electronic states across the whole energy level, emphasizing its superior electrical conductivity and metallic nature with Dirac-like characteristics (Fig. 8b). Importantly the charge density distribution provides observable insights into the electron-transfer tendency at the heterostructure interface. According to DFT analysis, there is an electron deficit at the edge of Bi_2_MoO_6_, whereas electrons primarily accumulate on the Ti_3_C_2_T_x_ side. This evidence corroborates the charge transfer of 0.12 *e*- from Bi_2_MoO_6_ to Ti_3_C_2_T_x_ within the Schottky barrier height of 0.45 eV, which simultaneously facilitates the reduction of electron reverse migration under piezoelectric polarization. Figure 8c, d shows density of states (DOS) plots to give a thorough understanding of the distributions of electronic energy levels and their corresponding orbital compositions in Bi_2_MoO_6_ and Ti_3_C_2_T_x_. The VB of Bi_2_MoO_6_ is primarily made up of hybridized Bi 6p and O 2p orbitals, whereas CB is primarily made up of hybridized O 2p and Mo 4 d orbitals (Fig. 8c). Significant conductivity of Ti_3_C_2_T_x_ as a cocatalyst is demonstrated by the continuous electronic states of the Ti 3 d, O 2p, and C 2p orbits across the Fermi level in the partial DOS of Ti_3_C_2_T_x_ (Fig. 8d), which is in line with improved piezo-electrochemical reactions. It is clear that O atoms at the interface between Bi_2_MoO_6_ and O-terminal Ti_3_C_2_T_x_ function as charge channels to promote electron transfer because of the critical role that O 2p orbitals play in the energy band edge of Bi_2_MoO_6_ and Ti_3_C_2_T_x_. This interfacial interaction is crucial in initiating water adsorption, a prerequisite for the hydrogen evolution reaction (HER). As illustrated in Fig. 8e, the Bi_2_MoO_6_/Ti_3_C_2_T_x_ heterostructure (−2.18 eV) exhibits a higher adsorption capability than Bi_2_MoO_6_ (−0.85 eV) and Ti_3_C_2_T_x_ (−1.62 eV), suggesting a greater tendency for adsorption and the provision of additional reaction sites on the catalyst surface. Additionally, a key indicator of different catalysts for H_2_ evolution activity is the Gibbs free energy for H atomic absorption $|\Delta {G}_{H}^{o}|$. Metal Pt is frequently used as a reference material, and a value near zero (0.12 eV) indicates a more favorable reaction. As illustrated in Fig. 8f, we computed $|\Delta {G}_{H}^{o}|$ for Bi_2_MoO_6_, Ti_3_C_2_T_x_, and Bi_2_MoO_6_/Ti_3_C_2_T_x_ heterostructures in relation to Pt in this investigation. The Gibbs free energy of adsorption with water coverage (ML) on the surfaces of Bi_2_MoO_6_, Ti_3_C_2_T_x_, and Bi_2_MoO_6_/Ti_3_C_2_T_x_ heterostructure is displayed in Table S2. As depicted in Fig. 8f and summarized in Table S2, Bi_2_MoO_6_ shows a relatively high ${|\varDelta G}_{H* }|$ of 0.31 eV, implying weak H* binding and facile desorption. In contrast, Ti_3_C_2_T_x_ shows improved performance ($|\Delta {G}_{H}^{o}|$ $=\,0.18{eV}$), and the Bi_2_MoO_6_/Ti_3_C_2_T_x_ heterostructure achieves the lowest $|\Delta {G}_{H}^{o}|$ value of 0.09 eV at 0.50 ML water coverage. The Bi_2_MoO_6_/Ti_3_C_2_T_x_ heterostructure has the lowest $|\Delta {G}_{H}^{o}|$ across all coverages, as Table S2 demonstrates, suggesting that the heterostructure has synergistic effects that improve the stability of water adsorption. The $|\Delta {G}_{H}^{o}|$ of heterostructure at various coverages are even significantly lower than the benchmark catalyst, Pt, confirming that adding Ti_3_C_2_T_x_ and a Schottky junction significantly increases H_2_ evolution efficiency. DFT calculations suggest good interface stability of Bi_2_MoO_6_/Ti_3_C_2_T_x_ heterostructure with binding energy of −0.32 eV ${{{\AA }}}^{-2}.\,$Overall, the electron-hole recombination can be suppressed and electron separation accelerated by the optimized electron transport channels at the interface of Bi_2_MoO_6_/Ti_3_C_2_T_x_. To a certain degree, highly efficient piezocatalytic H_2_ evolution can be ensured by simultaneously attracting more H_2_O molecules to the catalyst surface for a reduction reaction with optimized H_2_O adsorption Gibbs free energy and lower H atomic activation energy.

**Fig. 9:**
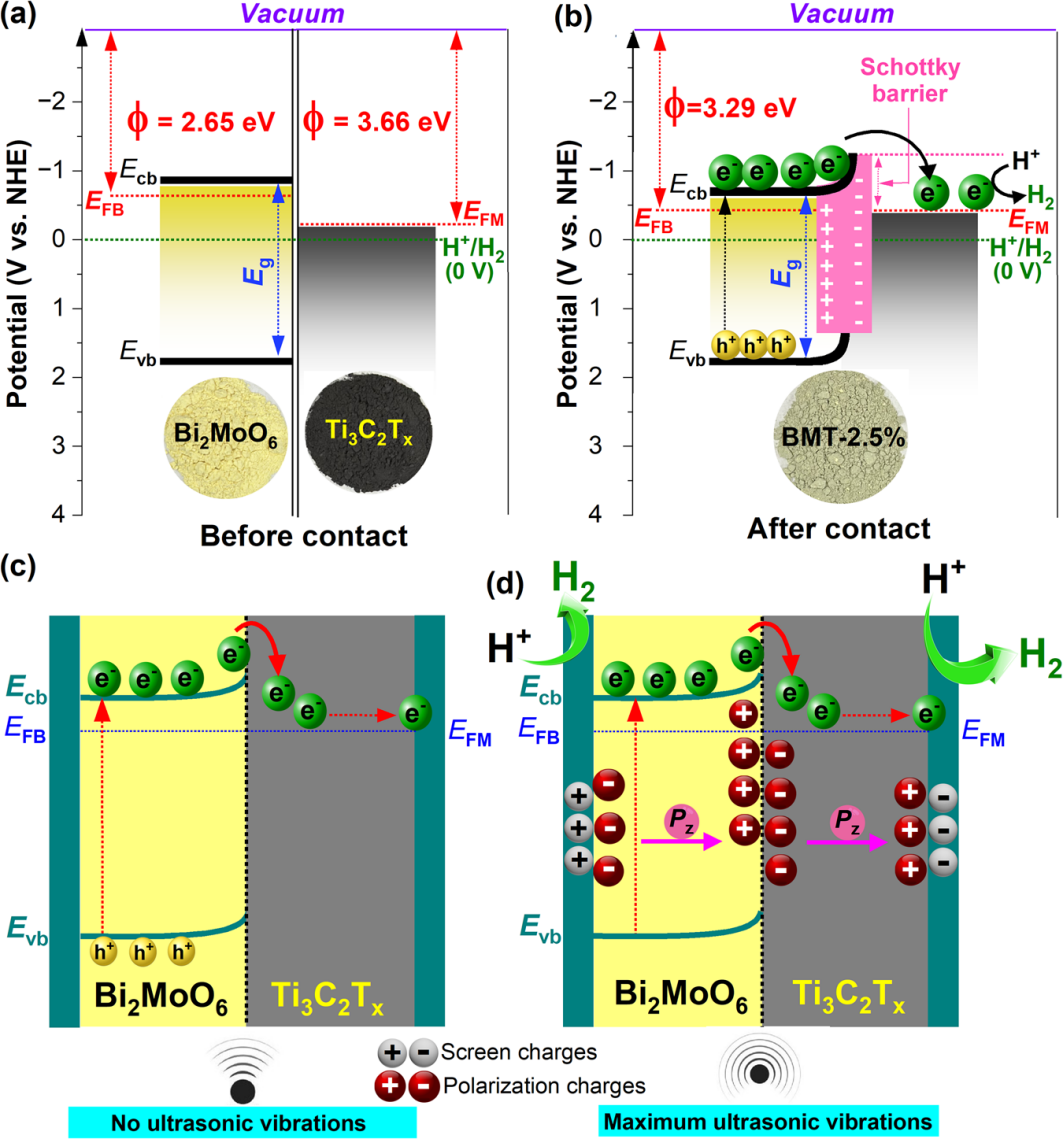
Proposed formation and piezocatalytic mechanism of the Bi_2_MoO_6_/Ti_3_C_2_T_x_ Schottky junction. **a**, **b** The formation mechanism models of Bi_2_MoO_6_/Ti_3_C_2_T_x_ Schottky junction. Proposed schematic illustration of the piezocatalytic hydrogen evolution mechanism in Bi_2_MoO_6_/Ti_3_C_2_T_x_ under ultrasonic excitation. **c** In the absence of ultrasonic vibrations, the Bi_2_MoO_6_/Ti_3_C_2_T_x_ system remains in an electrically neutral state. **d** Interfacial redox processes are driven by significant driving forces, which release active electrons and increase negative polarization charges during ultrasonic vibration. The generated polarization charges and corresponding screening charges reach a dynamic equilibrium, enabling charge carrier redistribution and prolonged catalytic activity.

Based on the insights from UPS analysis, Tauc plot-derived optical band gaps, XPS valence band analysis, and DFT calculations, a mechanistic model is proposed to illustrate the role of the Bi_2_MoO_6_/Ti_3_C_2_T_x_ Schottky junction in facilitating piezocatalytic H_2_ evolution (Fig. 9a–d). Upon contact between Bi_2_MoO_6_ and Ti_3_C_2_T_x_, electrons migrate from Bi_2_MoO_6_ to Ti_3_C_2_T_x_ due to the difference in their work functions (ΦTi_3_C_2_T_x_ > ΦBi_2_MoO_6_), resulting in Fermi level equilibration and the formation of an interfacial Schottky barrier. This band alignment induces an upward bending of the conduction and valence bands in Bi_2_MoO_6_. Under equilibrium conditions and without external mechanical excitation through ultrasonic vibrations, H_2_ evolution is insignificant (Fig. 9c), primarily due to the low concentration and rapid depletion of free charge carriers on the Bi_2_MoO_6_/Ti_3_C_2_T_x_ surface when immersed in aqueous phase. However, when subjected to maximum ultrasonic vibration, cavitation-induced bubbles collapsed and generated transient high-strain fields (~10^8 ^Pa),^74^ acting as a strong driving force for piezocatalytic redox reactions on the Bi_2_MoO_6_/Ti_3_C_2_T_x_ surface (Fig. 9d). This mechanical perturbation excites electrons from the valence band to the conduction band in Bi_2_MoO_6_, initiating charge carrier generation. Owing to the excellent electrical conductivity of Ti_3_C_2_T_x_, these piezogenerated electrons preferentially transfer from Bi_2_MoO_6_ to Ti_3_C_2_T_x_ surface, enhancing charge separation. Simultaneously, the vibration-induced piezoelectric effect in Bi_2_MoO_6_ generates a built-in electric field and substantial piezoelectric potential (*P*_z_), facilitating the directional migration of electrons and holes in opposite directions within the Bi_2_MoO_6_/Ti_3_C_2_T_x_ surface. The alignment of polarization charges further amplifies the local electric field, drawing screened charges to the regions of excess polarization and modulating the efficiency of surface catalytic reactions. The accumulation of electrons on the Ti_3_C_2_T_x_ surface, coupled with negative polarization charge density, effectively drives the reduction of protons to molecular H_2_, thereby enhancing the overall piezocatalytic performance. This dynamic carrier redistribution under ultrasonic excitation highlights the vital role of the piezoelectric potential in sustaining the catalytic cycle within the Bi_2_MoO_6_/Ti_3_C_2_T_x_ Schottky junction. Collectively, these findings not only advance the mechanistic understanding of Schottky junction-mediated piezocatalysis but also establish a versatile strategy that combines cocatalyst engineering and interfacial Schottky design. This strategy holds significant promise for expanding the application of MXene-based materials in diverse piezocatalytic energy conversion processes, including N_2_ fixation, CO_2_ reduction, and H_2_O_2_ synthesis in near future.

## Conclusions

In summary, we have successfully constructed a Bi_2_MoO_6_/Ti_3_C_2_T_x_ Schottky junction that enables efficient interfacial electron transport for enhanced piezocatalytic hydrogen evolution. The optimized Bi_2_MoO_6_/Ti_3_C_2_T_x_ system harnesses the synergistic interaction between Schottky barrier formation and piezoelectric polarization fields to significantly improve charge carrier separation, facilitate directional charge migration, and create additional reactive sites. These collective effects result in superior piezocatalytic hydrogen evolution activity and long-term operational stability and durability of the synthesized piezocatalysts, outperforming other reported piezocatalytic systems. Comprehensive experimental and computational analysis further reveal that the Bi_2_MoO_6_/Ti_3_C_2_T_x_ Schottky junction modulates the electronic structure of the catalyst, thereby promoting charge separation and effectively suppressing electron–hole recombination during the catalytic process. This work not only deepens the mechanistic understanding of MXene-based piezocatalysts but also provides a versatile strategy for the rational design of high-performance materials via Schottky junction construction and cocatalyst optimization. The insights presented here open new avenues for advanced piezocatalytic applications, including sustainable hydrogen production and broader energy conversion technologies.

## Methods

### Synthesis of Bi_2_MoO_6_ microspheres

To synthesize 3D hierarchical Bi_2_MoO_6_ microspheres, 0.85 g of Bi(NO_3_)_3_.5H_2_O and 0.2 g of Na_2_MoO_4_.7H_2_O were each dissolved separately in 5 mL of ethylene glycol under magnetic stirring at 60 °C. Following complete dissolution, the Na_2_MoO_4_.7H_2_O solution was gradually introduced into the Bi(NO_3_)_3_.5H_2_O solution under continuous stirring. Subsequently, 25 mL of absolute ethanol was slowly added to the mixed solution, and the mixture was stirred for an additional 15 minutes to ensure homogeneity. The resulting 35 mL solution was transferred into a 100 mL Teflon-lined stainless-steel autoclave and subjected to hydrothermal treatment at 160 °C for 6 hours, followed by natural cooling to ambient temperature. The solid product was collected via filtration and washed repeatedly (≥6 times) with absolute ethanol and deionized water to eliminate residual impurities, followed by overnight drying at 80 °C. Final high-purity 3D Bi_2_MoO_6_ microspheres were collected by grinding the dried powder using an agate mortar for 30 minutes.

### Synthesis of nanolayered Ti_3_C_2_T_x_ sheets

1 g of Ti_3_AlC_2_ MAX phase powder was gradually introduced into 20 mL of 9 M HCl containing 1 g of LiF under continuous stirring at 35 °C. The etching process was maintained for 24 h to selectively remove the Al atomic layers. The resulting suspension was subjected to centrifugation at 3500 rpm for 30 min, followed by repeated washing with deionized water until the supernatant reached a neutral pH (≥6), yielding multilayered Ti_3_C_2_T_x_. To synthesize nanolayered Ti_3_C_2_T_x_, the collected product was then re-dispersed in deionized water and ultrasonicated for 1 h in an ice bath under an Ar atmosphere to achieve exfoliation. After that, the dispersion underwent the same centrifugation and washing steps, and the final product was freeze-dried at -55 °C to obtain nanolayered Ti_3_C_2_T_x_ sheets.

### Synthesis of Bi_2_MoO_6_/Ti_3_C_2_T_x_ heterostructure

To fabricate the Bi_2_MoO_6_/Ti_3_C_2_T_x_ heterojunctions, a designated amount of nano-layered Ti_3_C_2_T_x_ sheets were uniformly dispersed in 50 mL of deionized water through a combination of magnetic stirring and ultrasonic treatment. Subsequently, 100 mg of Bi_2_MoO_6_ powder was added to the dispersion, and the mixture was stirred for 1 hour to promote electrostatic self-assembly of the heterostructure. The resulting composite was recovered via filtration, thoroughly washed with deionized water, and dried under vacuum at 12 °C for 12 h. By adjusting the loading of Ti_3_C_2_T_x_, mass ratios of 0.5%, 2.5%, and 5.0% relative to Bi_2_MoO_6_ were achieved. The corresponding samples were designated as BMT-0.5%, BMT-2.5%, and BMT-5.0%, respectively.

### Piezocatalytic hydrogen generation experiments

In a typical piezo-catalytic hydrogen evolution experiment, 5 mg of the as-prepared material was dispersed in 100 mL of a mixed solvent consisting of deionized water and methanol (MeOH) in a 4:1 volume ratio. The reaction system was assembled in a 250 mL borosilicate headspace conical flask and then purged with high-purity nitrogen (N_2_) gas for 30 min to establish an inert atmosphere. The flasks were then sealed with Suba-Seal rubber septa (Sigma-Aldrich) to maintain an anaerobic environment. An ultrasonic cleaner operating at 35 kHz and 120 W was employed as the mechanical excitation source to initiate the piezo-catalytic process. Gas samples were collected at regular intervals using a 10 mL high-precision gas-tight syringe (Pressure-Lok®, VICI Valco Instruments, USA) and analyzed using gas chromatography (GC-Clarus 590, PerkinElmer, USA) equipped with a thermal conductivity detector (TCD). All experiments were performed in triplicate to ensure reproducibility, and the average values were reported. Throughout the reaction, a constant temperature (25 °C) was maintained in the ultrasonic bath to ensure the accuracy and consistency of the catalytic performance measurements.

## Supplementary information

Supplementary Material

## Data Availability

The data that support the findings of this study are available from the corresponding author upon request.
