# Supplementary material for: Schottky-driven interfacial design of Bi2MoO6/Ti3C2Tx heterostructure for boosted piezocatalytic hydrogen evolution
Source: Commun Mater. 2026 May 6;7(1):181. doi: 10.1038/s43246-026-01168-z (PMC13357206; doi:10.1038/s43246-026-01168-z)
Supplement: Supplementary file 1 — Supplementary Material [file 43246_2026_1168_MOESM1_ESM.docx]

**Supplementary Information**

**Schottky-Driven Interfacial Design of Bi_2_MoO_6_/Ti_3_C_2_T_x_ Heterostructure for Boosted Piezocatalytic Hydrogen Evolution**

**Rahil Changotra**^a^**, Jie Yang**^b^**, Mita Dasog**^c,d^**, Quan (Sophia) He**^a*^

*^a^ Department of Engineering, Faculty of Agriculture, Dalhousie University, Truro, NS B2N 5E3, Canada*

*^b^ Institute of Oceanography, College of Geography and Oceanography, Minjiang University, Fuzhou 350108, China*

*^c^ Department of Chemistry, Dalhousie University, Halifax, NS, B3H 4R2, Canada*

*^d^ Department of Civil and Resource Engineering, Dalhousie University, Halifax, NS, B3H 4R2, Canada*

*Corresponding Author:

Address: *Professor, Department of Engineering, Faculty of Agriculture, Dalhousie University, 39 COX Road, Banting Building, Truro, NS B2N 5E3, Canada.*

E-mail: *quan.he@dal.ca*

Tel: +1-(902) 893.6180; Fax: +1 (902) 893-1859

**Text S1. Chemicals and reagents**

Bismuth nitrate pentahydrate (Bi(NO_3_)_3_.5H_2_O, ACS, ≥98.0%), sodium molybdate dihydrate (Na_2_MoO_4_.7H_2_O, ACS, ≥99.5%), lithium fluoride (LiF, <10 µm, ≥99.98%), ethylene glycol (anhydrous, 99.8%), sodium sulfite (Na_2_SO_3,_ ACS, ≥99.0%), sodium sulfate (Na_2_SO_4_, ACS, ≥99.0%), triethanolamine (TEOA, ACS, ≥99.0%), urea (ACS, ≥99.0%), glucose (99.5%), ethanol (EtOH anhydrous), and methanol (MeOH, HPLC grade, ≥99.9%) were purchased from Sigma-Aldrich. Ti_3_AlC_2_ MAX phase was procured from Nanochemazone, Canada. Sodium hydroxide (ACS, 97%), nitric acid (ACS, 68-70%), hydrochloric acid (AR, 36−38%), and the other chemical reagents were purchased from Fisher Chemicals. Deionized (DI) water was used throughout the study, which was collected from the water purification system (Direct-Q® Milli-Q, Millipore Sigma, Germany).

**Text S2. Material characterizations**

Morphology and microstructures of synthesized materials were determined using scanning electron microscopy (FE-SEM, FEI Quanta 450) equipped with energy dispersive X-ray spectroscopy (EDS) elemental mapping analysis, transmission electron microscopy (TEM, Talos F200E, Thermo Fisher Scientific, USA), high-resolution transmission electron microscopy (HRTEM, TFS Spectra Ultra, Thermo Fisher Scientific, USA), and atomic force microscopy (AFM, Bioscope Catalyst, Bruker). The distribution and clear distinction of elements was further confirmed using the high-angle annular dark-field (HAADF) imaging in scanning transmission electron microscopy (STEM, Talos F200X, Thermo Fisher Scientific, USA). The phase structure of the synthesized materials was analyzed using a powder X-ray diffractometer (Bruker D8 Advance) equipped with a *Cu Kα* radiation source (*λ* = 0.15406 nm). Raman spectroscopy was performed with excitation provided by 633 nm Ar^+^ laser (Renishaw, inVia Reflex). Fourier transform infrared (FT-IR) spectra were recorded using an FT-IR spectrometer (UATR Spectrum Two, PerkinElmer, USA) with a spectral resolution of $4 cm⁻¹$ over the range of $450-4000 cm⁻¹$. Diffuse reflectance spectra (DRS) of the synthesized materials were obtained using a UV-Vis-NIR spectrophotometer (Persee Instruments, China) over the wavelength range of 200-800 nm. The surface properties and bond energies were measured using X-ray photoelectron spectroscopy (XPS, Thermo Fisher Multilab 2000, USA) using an *Al Kα* radiation source (1486.6 eV) and pass energies of 30 eV for high-resolution scans and 100 eV for survey scans. The operational current and voltage were 20 mA and 10 kV, respectively. The XPS data analysis was conducted using CasaXPS software (version 2.3.23). The Shirley background and symmetric Gaussian/Lorentzian peak profiles were utilized for background subtraction and peak fitting, respectively. Ultraviolet photoemission spectroscopy (UPS) analysis was carried out using He I (21.22 eV) as a light source under a bias of $-10$ V (Thermo Scientific Nexsa G2 XPS) and the data was processed using Thermo Avantage (version 6.7.0). Zeta potentials were measured by a Zetasizer Nano ZS analyzer (Malvern Instruments). Photoluminescence measurements of solid materials were conducted using a fluorescence spectrometer (LS55, PerkinElmer, USA), at an excitation wavelength of 315 nm.

**Text S3: Piezo-response force microscopy (PFM) measurements and determination of piezoelectric coefficient**

The piezoelectric properties (amplitude-voltage butterfly and phase angle loops) were determined by piezo-response force microscopy (PFM, Dimension Icon, Bruker). Switching-spectroscopy piezoresponse force microscopy (SS-PFM) was employed to evaluate the local electromechanical response of Ti_3_C_2_T_x_ nanosheets and Bi_2_MoO_6_/Ti_3_C_2_T_x_ (BMT-2.5%) heterostructures. During SS-PFM measurements, a triangular DC bias waveform (up to ±10 V) was applied to generate amplitude-voltage (“butterfly”) and phase switching loops, while a small AC modulation voltage ($V_{\mathrm{ac}}$) was simultaneously applied for piezoresponse detection. It is important to note that the ±10 V corresponds to the DC bias sweep used to induce polarization switching, whereas $V_{\mathrm{ac}}$represents the small AC excitation voltage used for piezoresponse detection. The measured PFM amplitude signal was converted into out-of-plane surface displacement amplitude ($A_{\mathrm{disp}}$, pm) using instrument displacement calibration (deflection sensitivity). The effective piezoelectric coefficient ($d_{33}^{\mathrm{eff}}$) was estimated from the saturated responses of the two opposite polarization states using:^1^

$$d_{33}^{\mathrm{eff}}=\frac{A_{+}-A_{-}}{2V_{\mathrm{ac}}},$$

where $A_{+}$and $A_{-}$represent the displacement amplitudes at positive and negative saturation, respectively. Since electrostatic and non-switchable background contributions do not reverse with polarization, subtraction of the two saturated branches suppresses these artifacts and improves accuracy of the extracted piezoresponse. Back-calculation using calibrated displacement amplitudes indicates an AC modulation voltage of approximately 0.8 V, consistent with typical SS-PFM operating conditions.

**Text S4. Piezo-electrochemical measurements**

The piezo-electrochemical properties of the synthesized materials were evaluated using a 1010E Potentiostat (Gamry Instruments, USA) in a conventional three-electrode configuration. A platinum mesh served as the counter electrode, a saturated Ag/AgCl electrode was used as a reference, and the working electrode was prepared by modifying fluorine-doped tin oxide (FTO) glass substrates (sheet resistance of 7 Ω/sq). Prior to modification, FTO substrates were sequentially cleaned with absolute ethanol and deionized water. For electrode fabrication, 10 mg of the catalyst was dispersed in 100 μL of absolute ethanol via sonication for 30 minutes to form a uniform slurry. The suspension was drop-cast onto the cleaned and dried FTO substrate, covering an active area of 1 $cm^{2}$. The coated electrode was then air-dried at room temperature and subsequently annealed at 300 °C for 1 hour at 5 °C ${min}^{-1}$ rate to ensure proper adhesion and crystallinity. All photoelectrochemical measurements were conducted in a 0.5 M Na_2_SO_4_ aqueous electrolyte. Time-resolved current density measurements were carried out at a constant applied potential (1 V) versus the Ag/AgCl reference electrode, with the ultrasonic cleaner alternately switched on and off to assess the piezoelectric response under mechanical vibrational energy.

**Text S5. Computational Analysis** All density functional theory (DFT) calculations were carried out using the Vienna Ab initio Simulation Package (VASP). The electron-ion interactions were treated using the projector augmented-wave (PAW) method, and the exchange-correlation energy was described by the generalized gradient approximation (GGA) as implemented by the Perdew-Burke-Ernzerhof (PBE) functional.^2^ A plane-wave energy cutoff of 400 eV was employed to ensure the convergence of the total energy. Brillouin zone integrations were performed using the Gaussian smearing method with a smearing width of 0.05 eV, corresponding to ISMEAR = 0, and $2\times2\times1$ Monkhorst-Pack k-point sampling for structure. Our supercell was large enough to apply a Γ point sampling of the first Brillouin zone. Therefore, the self-consistent field (SCF) convergence criterion was set to $1\times{10}^{-8}$eV, and the relaxation of atomic structures was performed until the maximum force on each atom was less than 0.005 eV/Å. Static total energy calculations were executed using a fixed number of electronic steps (NELM = 100) with the blocked Davidson algorithm (IALGO = 38) for electronic minimization. In our structural calculations, we applied U correction for Ti 3d atoms (U = 3 eV) to reproduce the electronic structure.^3^ For both surfaces and interfaces, the vacuum gap perpendicular to the plane of the structure was fixed at 20 Å. A Bader analysis was performed to quantify charge transfer between the Bi_2_MoO_6_ and Ti_3_C_2_T_x_.^4^ Additionally, the following equations were used to compute the adsorption Gibbs free energy (Δ$G_{H}^{o}$), a crucial characteristic for H_2_ evolution:

$$E_{ads.}=E_{ad/sub}-E_{ad}-E_{sub}$$

Δ$G_{H}^{o}=\Delta E_{ads}+\Delta E_{ZPE}-T\Delta S_{H}$

where $E_{ad}$, $E_{ad/sub}$, and $E_{sub}$ represents the total energies of the adsorbate in the structure, optimized adsorbate/substrate system, and the clean substrate, respectively. Furthermore, Δ$G_{H}^{o}$, $T\Delta S_{H}$, $\Delta E_{ZPE}$, and $\Delta E_{ads}$ are the Gibbs free energy, entropic contributions, zero-point energy, and total energy from DFT calculations, respectively.


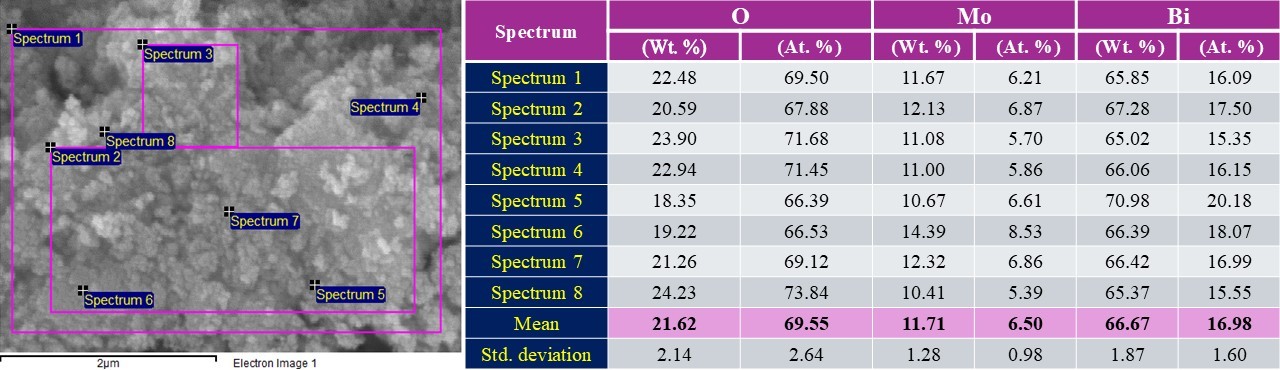


**Figure. S1** Elemental mapping of synthesized Bi_2_MoO_6_ microspheres.


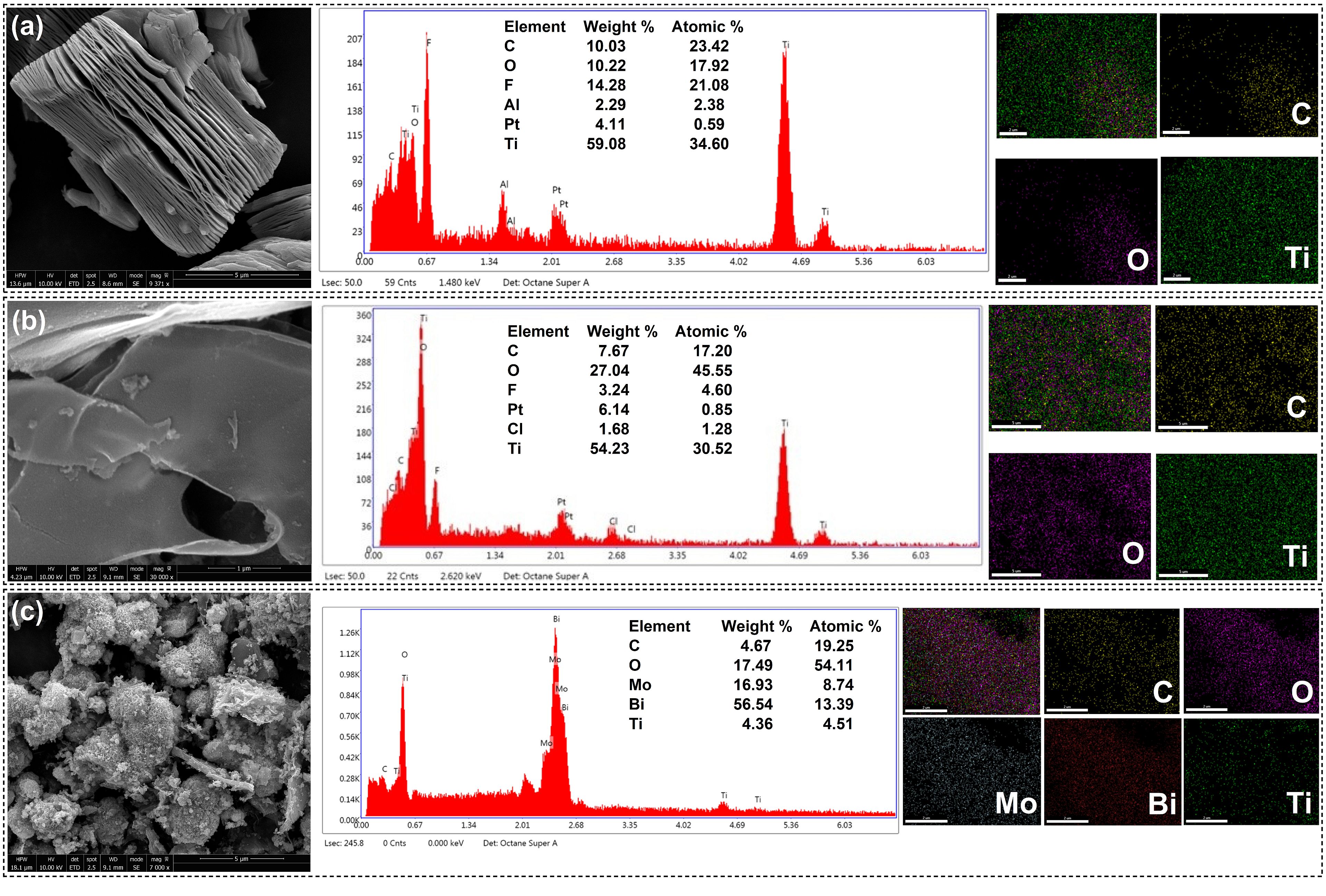


**Figure. S2** Elemental mapping of synthesized (a) multilayered Ti_3_C_2_T_x_, (b) nanolayered Ti_3_C_2_T_x_), and (c) Bi_2_MoO_6_/Ti_3_C_2_T_x_ heterostructure.


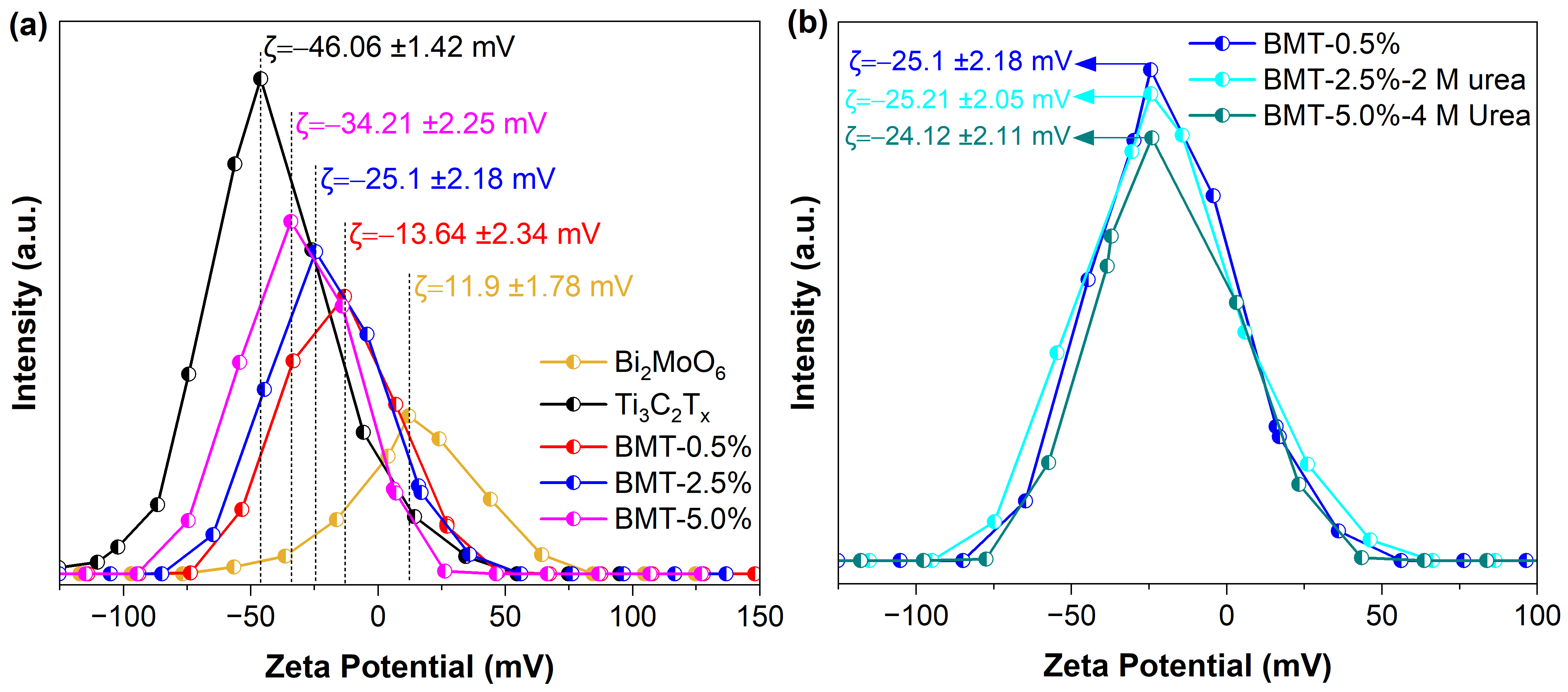


**Figure S3.** Zeta potential analysis of pristine Bi_2_MoO_6_, Ti_3_C_2_T_x_ MXene, and Bi_2_MoO_6_/ Ti_3_C_2_T_x_ heterostructures with different MXene loadings. **(a)** Progressive shift of ζ-potential from positive (Bi_2_MoO_6_) to increasingly negative values with higher MXene fractions, confirming electrostatic self-assembly at the interface. **(b)** Effect of urea addition (0–4 M) on ζ-potential of the composite, showing negligible change, thereby excluding hydrogen bonding as the dominant interaction mechanism.


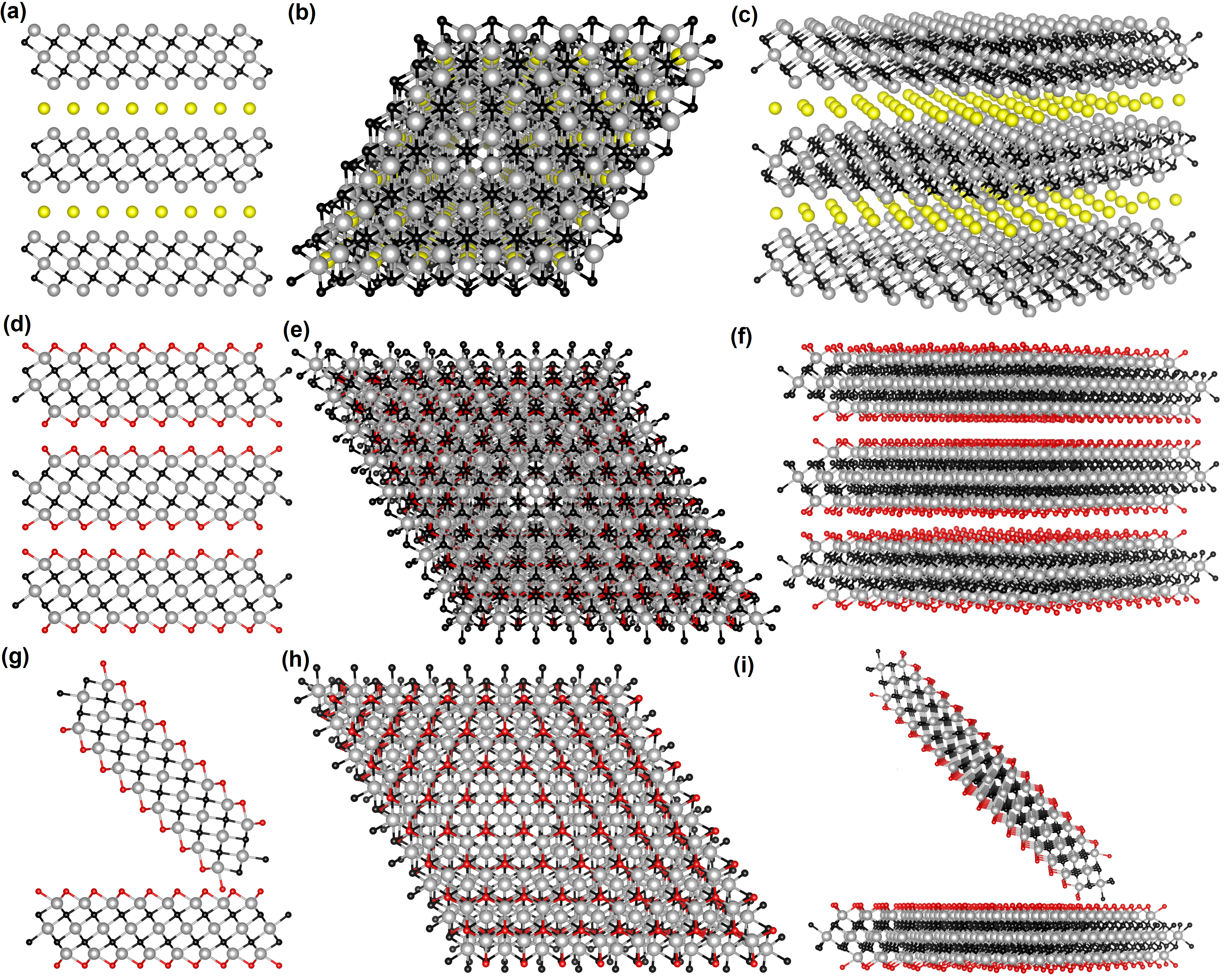


**Figure S4.** Top, side, and 3D isometric projections of atomic models of (a-c) Ti_3_AlC_2_ MAX phase, (d-f) multilayered Ti_3_C_2_T_x_, and (g-i) nanolayered Ti_3_C_2_T_x_.


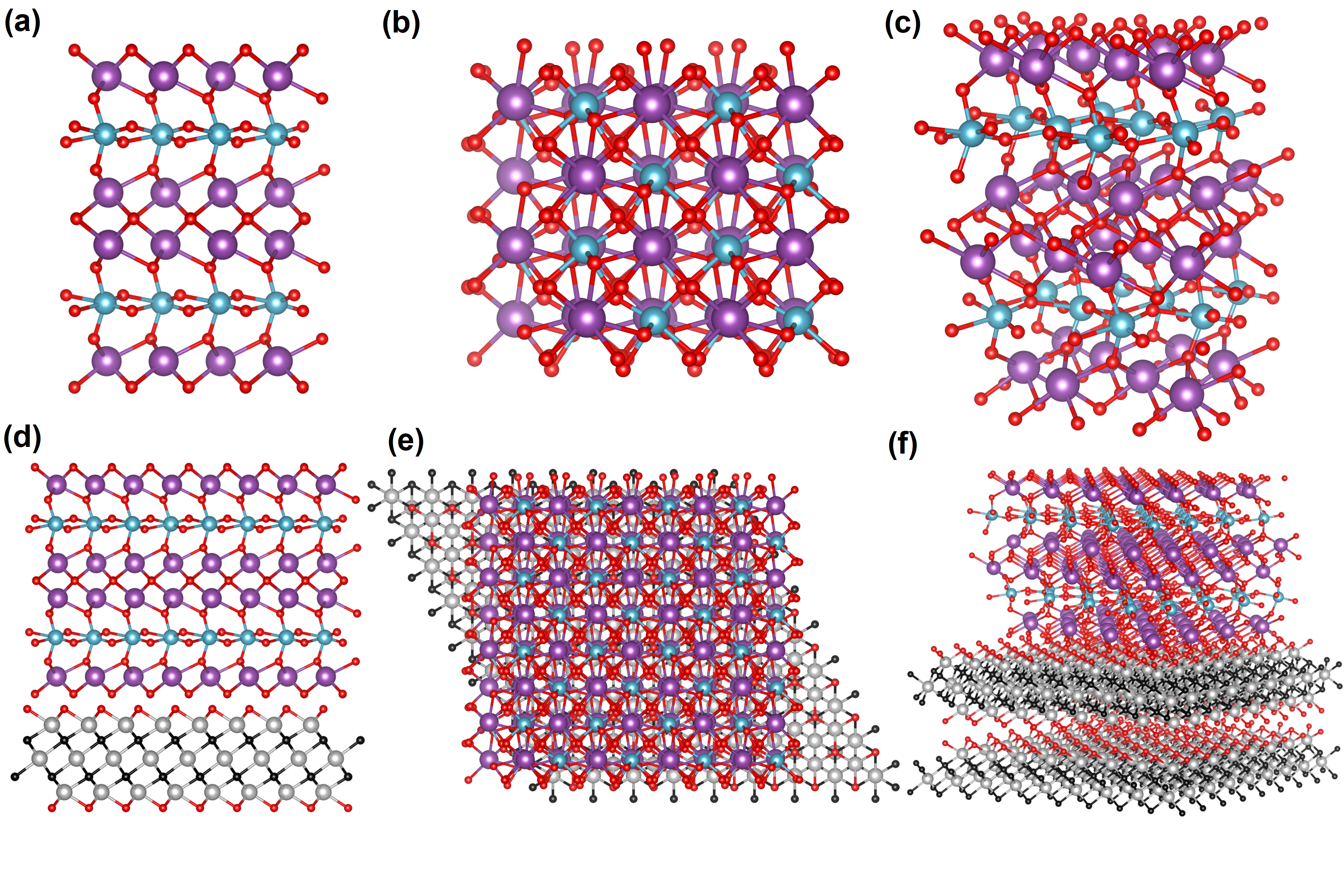


**Figure S5.** Top, side, and 3D isometric projections of atomic models of (a-c) Bi_2_MoO_6_, and (d-f) Bi_2_MoO_6_/Ti_3_C_2_T_x_ heterostructure.


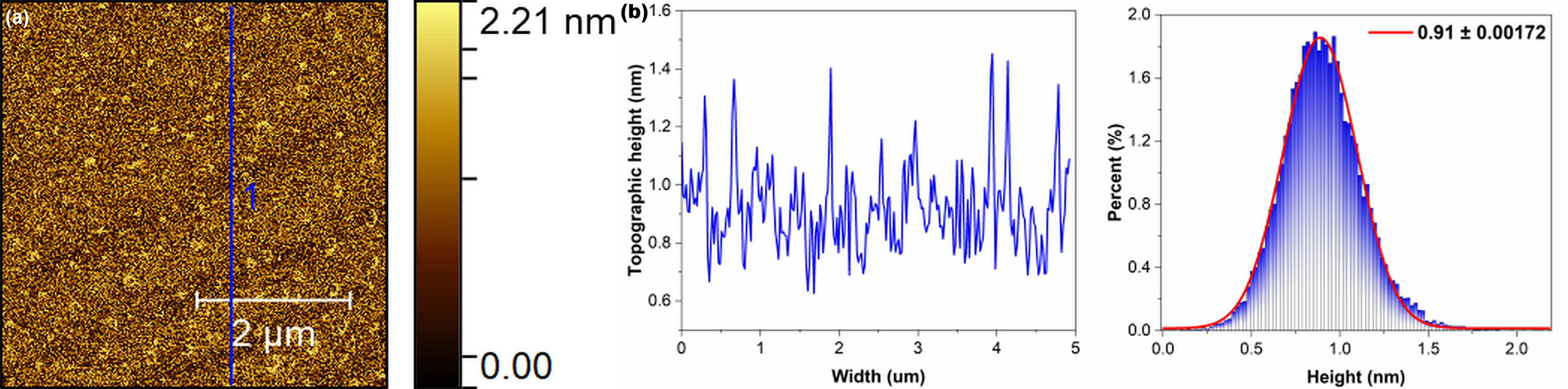


**Figure S6.** (a) AFM topography image of nanolayered Ti_3_C_2_T_x_ and (b) the corresponding lateral height measurement.


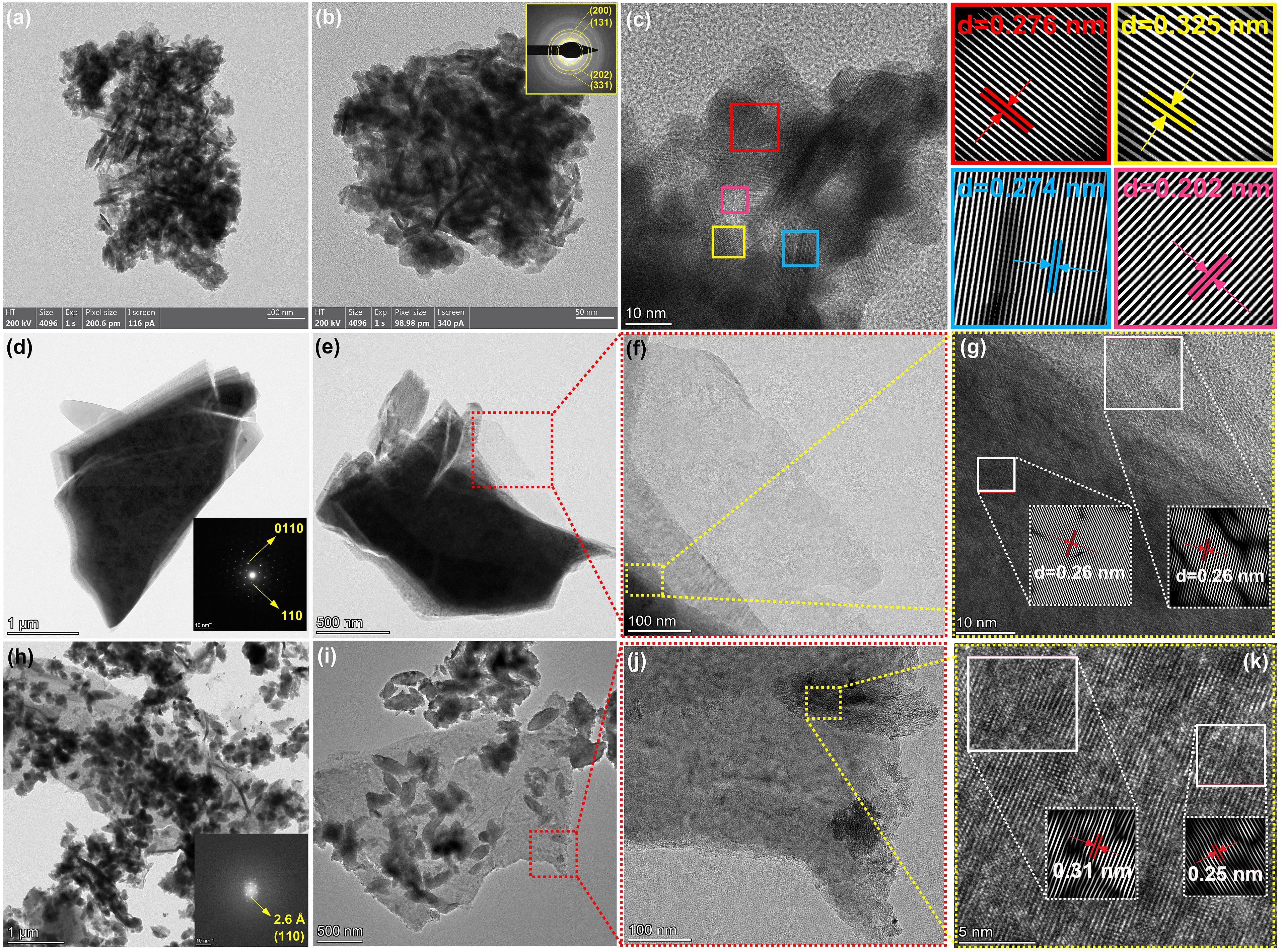


**Figure S7.** TEM and HRTEM (with d-spacing) of (a-c) Bi_2_MoO_6_, (d-g) multilayered Ti_3_C_2_T_x_ and (h-k) nanolayered Ti_3_C_2_T_x_.


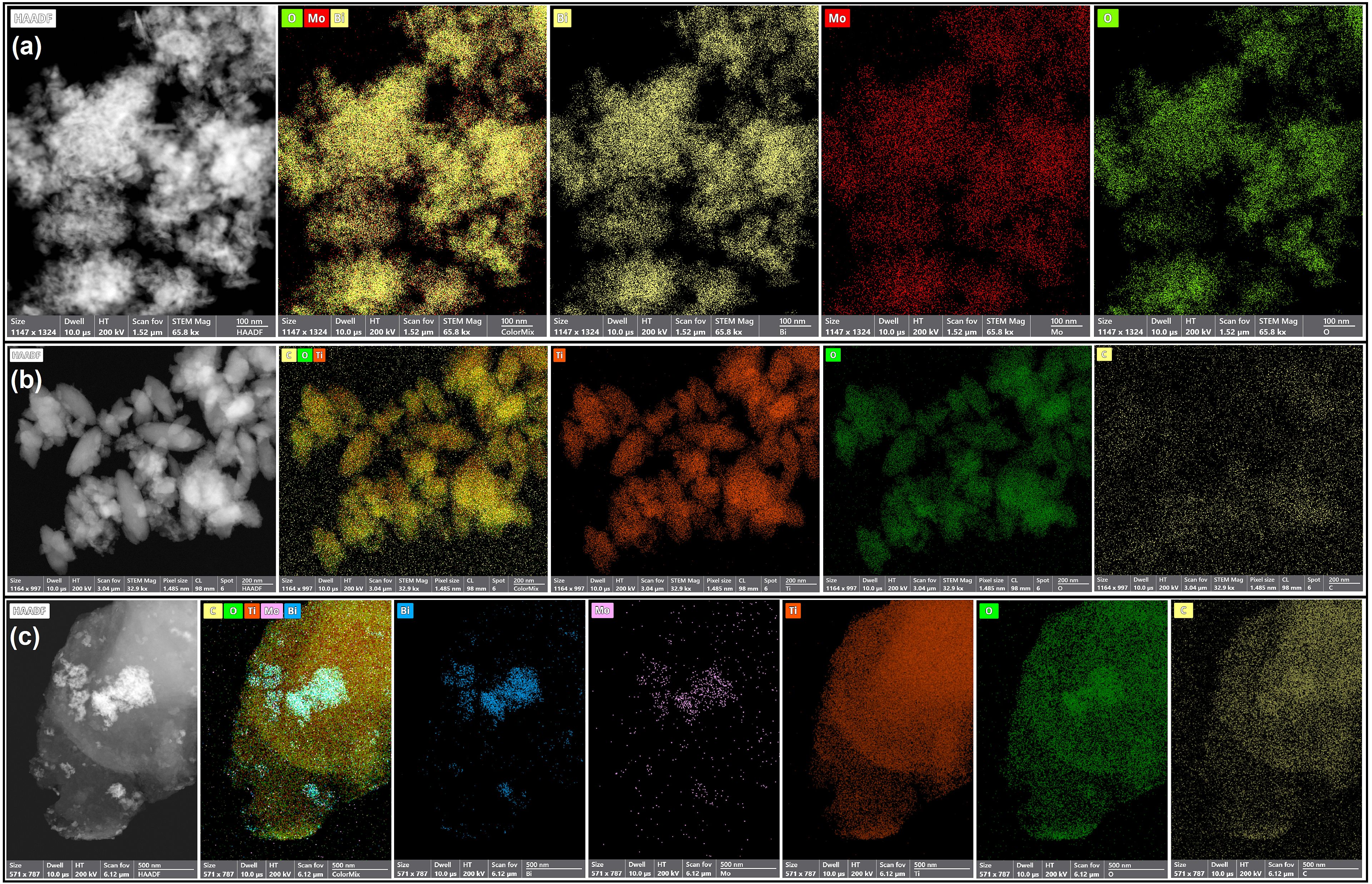


**Figure S8.** High-angle annular dark-field (HAADF)-STEM analysis of (a) Bi_2_MoO_6_, (b) Ti_3_C_2_T_x_ nanosheets, and (c) Bi_2_MoO_6_/Ti_3_C_2_T_x_ composite (BMT-2.5%).


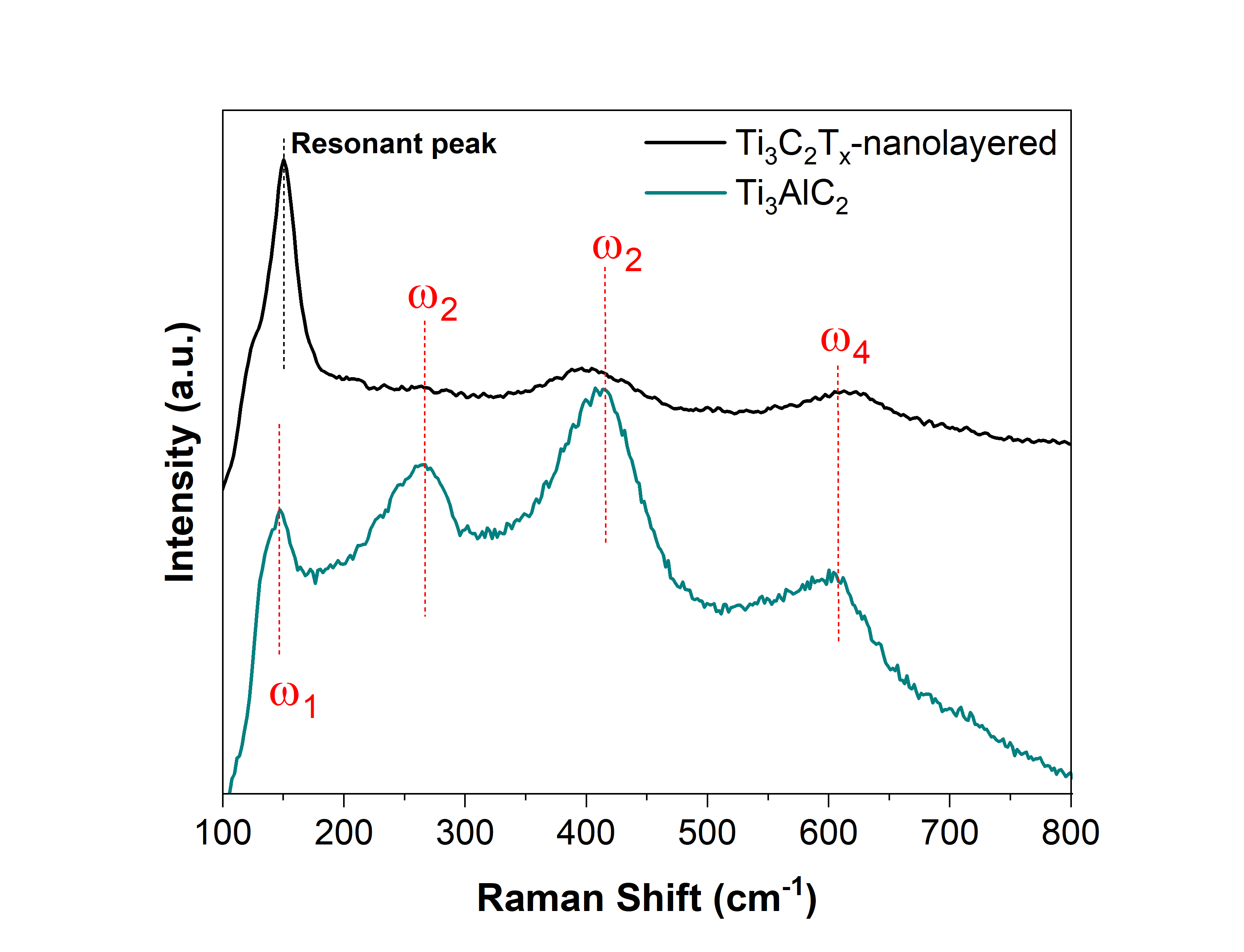


**Figure S9.** Enlarged curves of Raman spectra of Ti_3_AlC_2_ and Ti_3_C_2_T_x_.


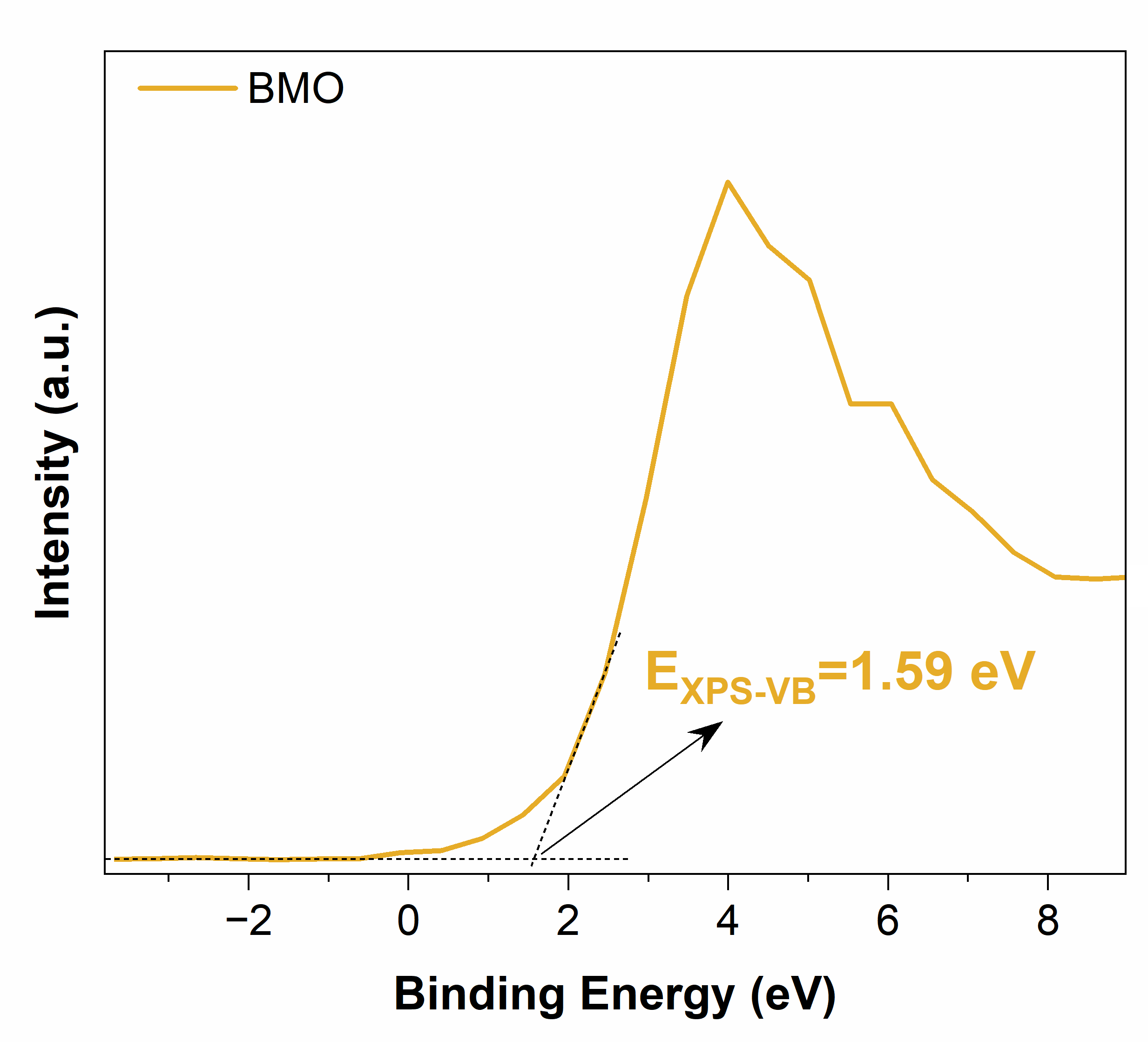


**Figure S10.** XPS-VB spectrum of Bi_2_MoO_6_.


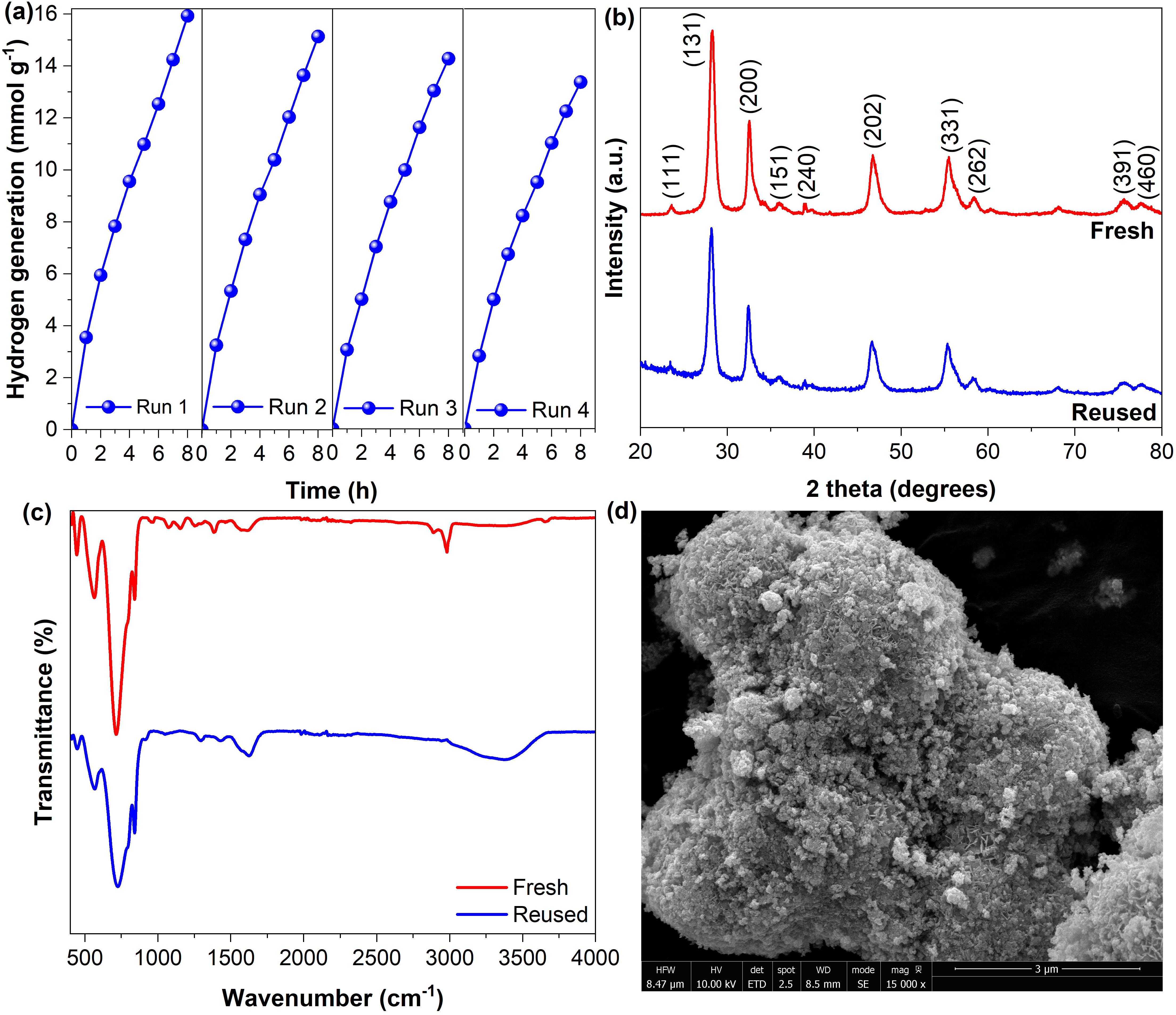


**Figure S11**. (a) Cyclic stability of BMT-2.5% for piezoelectric H_2_ evolution, (b) XRD comparison, and (c) FTIR comparison before and after the durability test, and (d) SEM image of BMT-2.5% after the durability test.


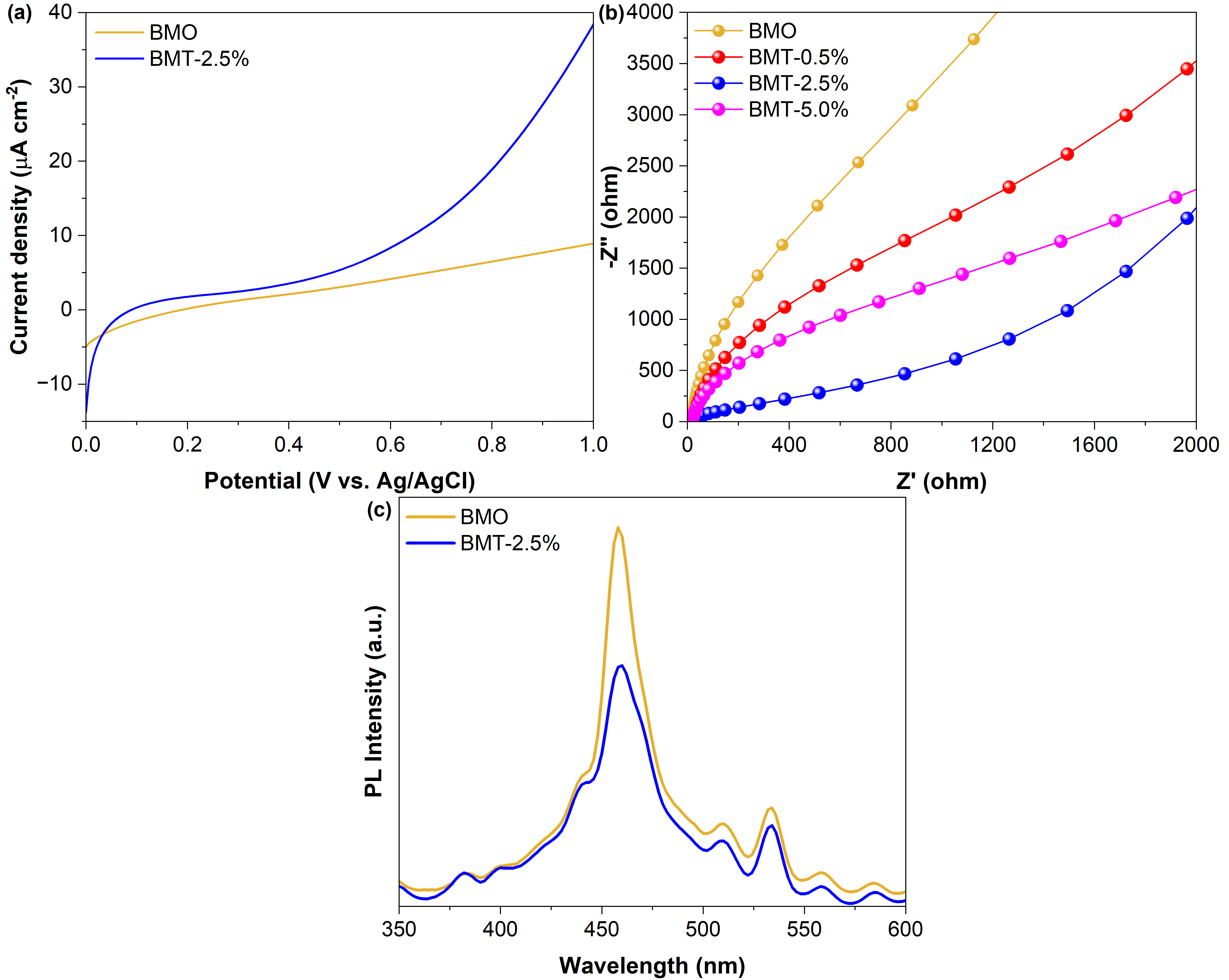


**Figure S12.** (a) Linear sweep voltammetry curves of Bi_2_MoO_6_ and BMT-2.5%. (b) Electrochemical impedance spectroscopy analyses of Bi_2_MoO_6_ and Bi_2_MoO_6_/Ti_3_C_2_T_x_ composites. (c) Photoluminescence spectra of Bi2MoO6 and BMT-2.5%.

**Table S1.** Piezocatalytic hydrogen evolution of various piezocatalyst.

| Piezocatalyst | Sacrificial agent | Ultrasonic vibrational energy | H_2_ evolution rate  (mmol g^-1^ h^-1^) | Ref. |
| --- | --- | --- | --- | --- |
| Bi_2_MoO_6_/Ti_3_C_2_T_x_ | Methanol | 35 kHz and 120 W | 1.991 | **This work** |
| MXene/Bi_2_WO_6_ | Methanol | 40 kHz and 150 W | 0.764 | ^5^ |
| CdS/BiOCl | Methanol | 40 kHz and 120 W | 1.048 | ^6^ |
| Bi_2_WO_6_ | Triethanolamine | 40 kHz | 0.191 | ^7^ |
| Pt@Sn_0.97_Ag_0.03_S_2_ | Na_2_SO_3_ | 45 kHz | 0.399 | ^8^ |
| BaTiO_3_ | Methanol | 35 kHz and 180 W | 0.305 | ^9^ |
| MoS_2_ | FeSO_4_ | 40 kHz and 100 W | 0.029 | ^10^ |
| UiO-66(Zr)-F4 | Na_2_SO_3_ | 40 kHz and 110 W | 0.178 | ^11^ |
| BiFeO_3_ | Na_2_SO_3_ | 60 kHz and 100 W | 0.124 | ^12^ |
| PbTiO_3_@CdS | Na_2_S/Na_2_SO_3_ | 40 kHz | 0.400 | ^13^ |
| 0.7BiFeO_3_/0.3BaTiO_3_ | Methanol | 40 kHz and 100 W | 1.322 | ^14^ |
| Sr_0.5_Ba_0.5_Nb_2_O_6_/Sr_2_Nb_2_O_7_ | Triethanolamine | 40 kHz and 110 W | 0.109 | ^15^ |
| Ba(Ti_0.89_Sn_0.11_O_3_)@Ag | Triethanolamine | 40 kHz and 120 W | 0.360 | ^16^ |
| C-doped KNbO_3_ | Triethanolamine | - | 0.524 | ^17^ |
| 2D SnSe | Triethanolamine | 45 kHz and 100 W | 0.948 | ^18^ |
| CdS | Na_2_S/Na_2_SO_3_ | 40 kHz and 150 W | 0.157 | ^19^ |
| Bi_0.5_Na_0.5_TiO_3_ | CH_3_OH | 40 kHz | 0.38 | ^20^ |
| Au–Bi_4_Ti_3_O_12_ | DI water | 40 kHz and 100 W | 0.194 | ^21^ |
| O-doped MoS_2_ | DI water | 40 kHz and 100 W | 0.047 | ^22^ |
| BiOCl | Methanol | 40 kHz and 120 W | 0.975 | ^23^ |
| La_2_NiO_4_ | DI water | 40 kHz and 100 W | 0.68 | ^24^ |
| GaN | Triethanolamine | 40 kHz and 110 W | 0.088 | ^25^ |
| PVDF-HFP | Methanol | - | 0.217 | ^26^ |
| V-doped NaNbO_3_ | DI water | 68 kHz and 192 W | 0.346 | ^27^ |
| Au/AgNbO_3_ | FeSO_4_ | 40 kHz and 110 W | 0.392 | ^28^ |
| BiOBr nanosheets | DI water | 40 kHz | 0.813 | ^29^ |
| BiOCl nanosheets | DI water | 40 kHz | 0.766 | ^29^ |
| BiOI nanosheets | DI water | 40 kHz | 0.518 | ^29^ |
| BiOBr nanosheets | Methanol | 40 kHz | 2.651 | ^29^ |
| Bi_4_TaO_8_Cl nanoplates (Sillen-Aurivillius) | DI water | 37 kHz and 110 W | 1.5 | ^30^ |
| Bi_4_O_5_Br_2_ nanosheets | DI water | 4 kHz and 240 W | 1.149 | ^31^ |
| Bi_4_O_5_I_2_ nanosheets | DI water | 4 kHz and 240 W | 0.764 | ^31^ |
| BiFeO_3_@COF | DI water | 40 kHz and 180 W | 0.180 | ^32^ |
| Bi_2_Fe_4_O_9_ nanoplates | DI water | 40 kHz and 200 W | 1.058 | ^33^ |

**Table S2.** Gibbs free energy of adsorption with water coverage (ML) on the surfaces of Bi_2_MoO_6_, Ti_3_C_2_T_x_, and Bi_2_MoO_6_/ Ti_3_C_2_T_x_ heterostructure.

| Coverage (ML) | Gibbs free energy of water adsorption ($\boldsymbol{\vert\Delta}\boldsymbol{G}_{\boldsymbol{H}}^{\boldsymbol{o}}\boldsymbol{\vert}$) | | |
| --- | --- | --- | --- |
|  | **Bi_2_MoO_6_** | **Ti_3_C_2_T_x_** | **Bi_2_MoO_6_/ Ti_3_C_2_T_x_ heterostructure** |
| 0.25 | 0.31 | 0.18 | 0.12 |
| 0.50 | 0.28 | 0.15 | 0.09 |
| 1.00 | 0.35 | 0.21 | 0.04 |

**References**

1 Jesse, S., Baddorf, A. P. & Kalinin, S. V. Switching spectroscopy piezoresponse force microscopy of ferroelectric materials. *Applied Physics Letters* **88** (2006). <https://doi.org/10.1063/1.2172216>

2 Ćwieka, K. *et al.* Zero carbon footprint hydrogen generation by photoreforming of methanol over Cu/TiO_2_ nanocatalyst. *Chemical Engineering Journal* **474**, 145687 (2023). <https://doi.org/10.1016/j.cej.2023.145687>

3 Dudarev, S. L., Botton, G. A., Savrasov, S. Y., Humphreys, C. & Sutton, A. P. Electron-energy-loss spectra and the structural stability of nickel oxide: An LSDA+ U study. *Physical Review B* **57**, 1505 (1998). https://doi.org/10.1103/PhysRevB.57.1505

4 Henkelman, G., Arnaldsson, A. & Jónsson, H. A fast and robust algorithm for Bader decomposition of charge density. *Computational Materials Science* **36**, 354-360 (2006). <https://doi.org/10.1016/j.commatsci.2005.04.010>

5 Ning, X. *et al.* Construction of MXene/Bi_2_WO_6_ schottky junction for highly efficient piezocatalytic hydrogen evolution and unraveling mechanism. *Nano Letters* **24**, 3361-3368 (2024). <https://doi.org/10.1021/acs.nanolett.3c04959>

6 Hao, P. *et al.* Rational design of CdS/BiOCl S-scheme heterojunction for effective boosting piezocatalytic H_2_ evolution and pollutants degradation performances. *Journal of Colloid and Interface Science* **639**, 343-354 (2023). <https://doi.org/10.1016/j.jcis.2023.02.075>

7 Xu, X. *et al.* Harvesting vibration energy to piezo-catalytically generate hydrogen through Bi_2_WO_6_ layered-perovskite. *Nano Energy* **78**, 105351 (2020). <https://doi.org/10.1016/j.nanoen.2020.105351>

8 Tian, W. *et al.* Enhanced piezocatalytic activity in ion-doped SnS_2_ via lattice distortion engineering for BPA degradation and hydrogen production. *Nano Energy* **107**, 108165 (2023). <https://doi.org/10.1016/j.nanoen.2023.108165>

9 Tang, Q. *et al.* Enhanced piezocatalytic performance of BaTiO_3_ nanosheets with highly exposed {001} facets. *Advanced Functional Materials* **32**, 2202180 (2022). <https://doi.org/10.1002/adfm.202202180>

10 Li, S. *et al.* Few-layer transition metal dichalcogenides (MoS_2_, WS_2_, and WSe_2_) for water splitting and degradation of organic pollutants: Understanding the piezocatalytic effect. *Nano Energy* **66**, 104083 (2019). <https://doi.org/10.1016/j.nanoen.2019.104083>

11 Zhao, S. *et al.* Harvesting mechanical energy for hydrogen generation by piezoelectric metal–organic frameworks. *Materials Horizons* **9**, 1978-1983 (2022). <https://doi.org/10.1039/D1MH01973B>

12 You, H. *et al.* Harvesting the vibration energy of BiFeO_3_ nanosheets for hydrogen evolution. *Angewandte Chemie* **131**, 11905-11910 (2019). <https://doi.org/10.1002/ange.201906181>

13 Huang, X. *et al.* Insight into the piezo-photo coupling effect of PbTiO_3_/CdS composites for piezo-photocatalytic hydrogen production. *Applied Catalysis B: Environmental* **282**, 119586 (2021). <https://doi.org/10.1016/j.apcatb.2020.119586>

14 Sun, Y. *et al.* Hydrogen generation and degradation of organic dyes by new piezocatalytic 0.7BiFeO_3_–0.3BaTiO_3_ nanoparticles with proper band alignment. *ACS Applied Materials & Interfaces* **13**, 11050-11057 (2021). <https://doi.org/10.1021/acsami.1c01407>

15 Dai, J. *et al.* Enhanced piezocatalytic activity of Sr_0.5_Ba_0.5_Nb_2_O_6_ nanostructures by engineering surface oxygen vacancies and self-generated heterojunctions. *ACS Applied Materials & Interfaces* **13**, 7259-7267 (2021). <https://doi.org/10.1021/acsami.0c21202>

16 Zhao, Q. *et al.* Highly-efficient piezocatalytic performance of nanocrystalline BaTi_0.89_Sn_0.11_O_3_ catalyst with Tc near room temperature. *Nano Energy* **85**, 106028 (2021). <https://doi.org/10.1016/j.nanoen.2021.106028>

17 He, J. *et al.* C-Doped KNbO_3_ single crystals for enhanced piezocatalytic intermediate water splitting. *Environmental Science: Nano* **9**, 1952-1960 (2022). <https://doi.org/10.1039/D2EN00244B>

18 Li, S. *et al.* Mechanically induced highly efficient hydrogen evolution from water over piezoelectric SnSe nanosheets. *Small* **18**, 2202507 (2022). <https://doi.org/10.1002/smll.202202507>

19 Wang, J., Hu, C., Zhang, Y. & Huang, H. Engineering piezoelectricity and strain sensitivity in CdS to promote piezocatalytic hydrogen evolution. *Chinese Journal of Catalysis* **43**, 1277-1285 (2022). <https://doi.org/10.1016/S1872-2067(21)63976-1>

20 Liu, D. *et al.* High-performance piezocatalytic hydrogen evolution by (Bi_0.5_Na_0.5_)TiO_3_  cubes decorated with cocatalysts. *Ceramics International* **49**, 20343-20350 (2023). <https://doi.org/10.1016/j.ceramint.2023.03.158>

21 Lei, R. *et al.* Cocatalyst engineering to weaken the charge screening effect over Au–Bi_4_Ti_3_O_12_ for piezocatalytic pure water splitting. *Catalysis Science & Technology* **12**, 7361-7368 (2022). <https://doi.org/10.1039/D2CY01422J>

22 Lei, R. *et al.* Free layer-dependent piezoelectricity of oxygen-doped MoS_2_ for the enhanced piezocatalytic hydrogen evolution from pure water. *Applied Surface Science* **576**, 151851 (2022). <https://doi.org/10.1016/j.apsusc.2021.151851>

23 Long, Y., Xu, H., He, J., Li, C. & Zhu, M. Piezoelectric polarization of BiOCl via capturing mechanical energy for catalytic H_2_ evolution. *Surfaces and Interfaces* **31**, 102056 (2022). <https://doi.org/10.1016/j.surfin.2022.102056>

24 Ma, X. *et al.* Enhanced charge separation in La_2_NiO_4_ nanoplates by coupled piezocatalysis and photocatalysis for efficient H_2_ evolution. *Nanoscale* **14**, 7083-7095 (2022). <https://doi.org/10.1039/D2NR01202B>

25 Zhang, M., Zhao, S., Zhao, Z., Li, S. & Wang, F. Piezocatalytic effect induced hydrogen production from water over non-noble metal Ni deposited ultralong GaN nanowires. *ACS Applied Materials & Interfaces* **13**, 10916-10924 (2021). <https://doi.org/10.1021/acsami.0c21976>

26 Song, L., Sun, S., Zhang, S. & Wei, J. Hydrogen production and mechanism from water splitting by metal-free organic polymers PVDF/PVDF-HFP under drive by vibrational energy. *Fuel* **324**, 124572 (2022). <https://doi.org/10.1016/j.fuel.2022.124572>

27 Li, Y. *et al.* Robust route to H_2_O_2_ and H_2_ via intermediate water splitting enabled by capitalizing on minimum vanadium-doped piezocatalysts. *Nano Research* **15**, 7986-7993 (2022). https://doi.org/10.1007/s12274-022-4506-0

28 Li, S. *et al.* Remarkably enhanced photocatalytic performance of Au/AgNbO_3_ heterostructures by coupling piezotronic with plasmonic effects. *Nano Energy* **95**, 107031 (2022). <https://doi.org/10.1016/j.nanoen.2022.107031>

29 Li, H.-R. *et al.* High-performance piezocatalytic hydrogen evolution over bismuth oxyhalides with halogen-dependent piezoelectricity and surface activity. *Rare Metals*, 1-11 (2025). https://doi.org/10.1007/s12598-025-03297-9

30 Banoo, M. *et al.* Bi_4_TaO_8_Cl as a new class of layered perovskite oxyhalide materials for piezopotential driven efficient seawater splitting. *Nano Letters* **22**, 8867-8874 (2022). <https://doi.org/10.1021/acs.nanolett.2c02900>

31 Wang, C. *et al.* Polar Layered Bismuth‐Rich Oxyhalide Piezoelectrics Bi_4_O_5_X_2_ (X=Br, I): Efficient Piezocatalytic Pure Water Splitting and Interlayer Anion‐Dependent Activity. *Advanced Functional Materials* **33**, 2301144 (2023). <https://doi.org/10.1002/adfm.202301144>

32 Xu, M. L. *et al.* Piezo‐photocatalytic synergy in BiFeO_3_@COF Z‐scheme heterostructures for high‐efficiency overall water splitting. *Angewandte Chemie* **134**, e202210700 (2022). https://doi.org/10.1002/ange.202210700)

33 Du, Y. *et al.* High-efficient piezocatalytic hydrogen evolution by centrosymmetric Bi_2_Fe_4_O_9_ nanoplates. *Nano Energy* **104**, 107919 (2022). <https://doi.org/10.1016/j.nanoen.2022.107919>
